# Mechanism of the Generation of New Somatic Compatibility Groups within *Thanatephorus cucumeris* (*Rhizoctonia solani*)

**DOI:** 10.1264/jsme2.ME12214

**Published:** 2013-08-31

**Authors:** Ping Qu, Mary Grace B. Saldajeno, Mitsuro Hyakumachi

**Affiliations:** 1 College of Life Sciences, Shaanxi Normal University, 199 South Chang’an Road, Xi’an 710062, China; 2 Laboratory of Plant Pathology, Faculty of Applied Biological Sciences, Gifu University, 1–1 Yanagido, Gifu 501–1193, Japan

**Keywords:** *Rhizoctonia solani*, somatic hyphal interactions, somatic compatibility group (SCG), heterokaryosis, AFLP analysis

## Abstract

Single-basidiospore isolates (SBIs) were obtained from field isolates of *Thanatephorus cucumeris* (*Rhizoctonia solani*) AG-1 IC and AG-2-2 IV. Formation of distinctive tufts, a recognized feature of heterokaryon synthesis, was observed, and isolates derived from hyphal-tipped tuft hyphae were obtained following pairings between various strains. Three distinctive types of tufts were formed: the fibrous type of mating-compatible homokaryon-homokaryon (Hom-Hom) pairings, the sparse type between heterokaryon-homokaryon (Het-Hom) pairings originating from one parent, and the compact type between Het-Hom pairings originating from different parents. Amplified Fragment Length Polymorphism (AFLP) profile of fingerprints of these tuft isolates verified that they were all heterokaryotic. Because of heterokaryotic vigor, the growth and pathogenicity of the majority of tuft isolates increased compared with their contributing SBIs. New somatic compatibility groups (SCGs) that were different from parental field isolates occurred following heterokaryon formation within *T. cucumeris*. Tuft isolates produced by Hom-Hom and Het-Hom pairings among isolates of different parents yielded no somatic compatibility with the original parent isolates and a high frequency of new SCGs (62–100%). This was in contrast to those produced by Hom-Hom and Het-Hom pairings among isolates with a common parent that yielded only 12–37% new SCGs. The SCG diversity of *R. solani* in the field may be attributed to new fitter heterokaryons formed between a heterokaryon of one pair of parents and a homokaryon of another parent pair. This mechanism greatly contributes to genetic diversity in the field and accounts for the failure to recover the expected distribution of SCGs from a field population.

*Thanatephorus cucumeris* (Frank) Donk [anamorph: *Rhizoctonia solani* Kühn] is known as a soil-borne plant pathogen and causes economically important diseases in a large variety of vegetables, crop plants, turf grasses, ornamentals, fruits and forest trees worldwide (1, 6, 40). Although isolates from some of the anastomosis groups (AGs) are pathogenic, individual isolates within an AG often have a distinct host range and differ in virulence. For example, AG-3 has a narrow host range (restricted to solanaceous plants) and is mainly a pathogen of potato, whereas AG-4 has a broad host range and is destructive to a number of different economically important plants (6). Also, isolates of some of the AGs, such as AG-6, AG-9, AG-10, AG-12, and AG-13, are considered non-pathogenic (10–12, 27, 39). Besides its economic importance, *T. cucumeris* is an important research tool for basic aspects of physiology, ecology and genetics.

Because field isolates of the basidiomycetous *T. cucumeris* are usually heterokaryons (Het), similar to the dikaryons (Di) of most basidiomycetes, anastomosis reactions are assumed to correspond to heterokaryon-heterokaryon (Het-Het) pairings, in most instances similar to the traditional Di-Di pairings of more typical basidiomycetes (14, 22, 28, 29, 45). The types of somatic compatibility reactions in *R. solani* are defined by Carling (9) and divided into 4 types (C0–C3) where: C0 = no anastomosis reaction; C1 = somatic incompatibility where hyphal contact occurs, hyphal attachment is apparent, no membrane fusion occurs; C2 = somatic incompatibility where wall fusion is obvious, membrane fusion is probable, anastomosing cells frequently die; C3 = somatic compatibility where the wall and membrane of anastomosing cells fuse, anastomosing cells frequently remain alive. When the fusion reactions correspond to the C3 reaction, the two isolates are somatically compatible, belonging to the same somatic compatibility group (SCG). It is assumed that incompatible C1 and C2 reactions occur as a result of the evolution of a mechanism for the fungus to maintain individual identity (22, 45 and some references therein). In basidiomycetes, the individuals are mated N+N (haploid + haploid) organisms, unlike similar reactions among ascomycetous fungi where the assumed somatic compatibility occurs between haploid organisms (17). The somatic compatibility systems of Ascomycetes are usually referred to as vegetative compatibility systems (VCGs) (16, 25, 45) to help differentiate ascomycetous from basidiomycetous systems protecting individuality. Both basidiomycetous and ascomycetous somatic compatibility reactions are often observable macroscopically on agar media as distinctive zones of mycelial interaction (25, 45).

Somatic compatibility reactions occur between isolates of fungi with differing genetic characteristics and differing genetic systems governing compatibility. The reaction between hyphae of paired isolates from different AGs will form incompatible interactions (25, 45).

The field population of *T. cucumeris* has been found to consist of many AGs and many SCGs within AGs, based on hyphal fusion interactions. Formation of a demarcation line, also known as an interaction zone at the interface between paired colonies of different isolates within a species or anastomosis group, was studied in the basidiomycetes *Athelia* (*Sclerotium*) *rolfsii* (35) and *T. cucumeris* (2) as well as in the ascomycetes *Leucocytospora kunzei* (34) and *Leucostoma persoonii* (3). Paired isolates showing no demarcation line are identified as belonging to the same SCG (or VCG). On the other hand, paired isolates showing a clear demarcation line at the junction of two colonies are identified as belonging to different SCGs (or VCGs).

In the field, it is known that the same SCGs of *T. cucumeris* AG-1 IA are rarely present in the same area and SCGs differ greatly between two seasons (spring and autumn) within a year or between seasons in two consecutive years (20). Within an AG, the population biology has been found to consist of many SCGs based on barrage reactions in the interaction zones. The mechanism behind the generation of genetic diversity in these SCGs in the field has not been analyzed in detail.

Sexual genetics of several *T. cucumeris* AGs have been studied by heterokaryon formation, synonymous with dikaryon formation in other basidiomycetes. Homokaryotic isolates (synonymous to monokaryons) are normally derived from sexual spores (single basidiospores), and are used for observation of heterokaryotic formation. The formation of a heterokaryon can be detected by the production of tufts of aerial mycelia growing at the junction of two compatible single-basidiospore colonies (2, 5, 23, 44). So far, mating phenomena have been studied only in AG-1, −2-2 IV, −4 and −8. It is considered that field isolates of AG-1 IA and AG-2 are homokaryotic, while field isolates of AG-1 IC, AG-4 and AG-8 are heterokaryotic with a bipolar mating system (1, 2, 6, 23, 24, 46). In AG-2-2 IV, Toda and Hyakumachi (41) reported that both homothallic and heterothallic mating systems were observed, and that genetic exchange could occur between heterothallic and homothallic isolates. AG-1 IA also has both homothallic and heterothallic mating systems based on the study of single-protoplast isolates (36). Rosewich *et al.* (37) reported a high degree of gene flow between populations and regular outcrossing within field populations in AG-1 IA.

In this study, we utilized C2 and C3 reactions in the interaction zones between Het-Het pairings, and Het-Hom pairings (similar to Di-Mon pairings) to study the diversity of SCGs formed within *T. cucumeris* AG-1 IC and within AG-2-2 IV. Homokaryons were selected as SBIs after inducing field isolates in fruit. Their mating type was determined, paired with other SBIs (Hom-Hom pairings), and their parental heterokaryons or non-parental heterokaryons (Het-Hom pairings) to determine whether heterokaryosis results in the synthesis of isolates of particular SCGs or new SCGs. The formation of SCGs was studied to determine whether heterokaryosis has any relationship to population variation within AGs of *T. cucumeris*.

## Materials and Methods

### Isolates

Three field isolates (189, Rh28 and 1R4) of *T. cucumeris* AG-1 IC and three (SA-1, H10-28 and H10-268) of AG-2-2 IV were used for the production of single basidiospore isolates (SBIs) in this study (Table 1). All isolates were maintained on 9-cm Petri dishes containing 12 mL potato dextrose agar (PDA [Becton Dickinson and Company, Sparks, MD, USA]) or in test tubes at 25°C.

### Production of sexual state and isolation of SBIs

The soil-over-culture method was used to produce the perfect state of each field isolate (32). A single 3 to 5 mm agar plug of each field isolate from stock culture was inoculated onto a 9-cm Petri dish containing 10 mL PDA at 25°C in the dark. After 3 days, a 5 mm agar plug with actively growing mycelium was cut from the edge of the hyphal colony and placed in the center of a new 9-cm Petri dish containing 30 mL PDYA (PDA containing 2.5% Bacto Yeast Extract [Becton Dickinson and Company, USA]) at 25°C in the dark. When the mycelia overspread the PDYA medium after 4 to 5 days’ incubation, sterilized soil blocks used for seedling culture of rice (Kureha Chemical Industry, Tokyo, Japan) were placed on the cultures covering the mycelia. Mycelia covered with soil blocks were incubated in a growth chamber at 28–30°C for 5 to 7 days in the dark. Sporulation occurred readily under high humidity conditions. Distilled water was added twice per day to maintain soil humidity. When evidence of sporulation showed on the surface of soil blocks, a piece of soil containing spores was picked up with sterile forceps and placed on a glass slide. A piece of soil was stained with 0.05% cotton blue to examine basidia and basidiospore formation by microscopy at 400× magnification. When basidia and basidiospores were observed, plates with soil blocks were inverted over Petri dishes containing acidified water agar with added 3% lactic acid (pH 4.0) (AWA). After 2 to 6 days, hyphae germinated from basidiospores were observed with a stereoscopic microscope. A hyphal tip germinated from each spore was picked with a sterilized spatula and transferred onto a Petri dish containing 10 mL PDA and saved as SBI. Approximately 50 SBIs obtained from each field isolate were assigned an Arabic number and an asterisk, *i.e.*, 1*, 2*, 3*, and so on and saved for further study (Table 1).

### Pairing incubation between field isolates and their SBIs

Pairing incubations among field isolates of AG-1 IC and their SBIs were performed using PDA with 0.5% activated charcoal (PDCA), a modified method of Julian *et al.* (23). For AG-2-2 IV, PDA with 2% activated charcoal was used (41). Selected SBIs from each of the field isolates (189, Rh28 and 1R4) of AG-1 IC and AG-2-2 IV (SA-1, H10-28 and H10-268) (Table 1) were paired with their parental and non-parental field isolates in all possible combinations of Hom-Hom, Het-Hom, and Het-Het pairings. Five millimeter PDA agar plugs of each isolate were placed 3 cm apart in a 9 cm Petri dish containing 20 mL PDCA. After 3–4 days’ incubation at 25°C in the dark, when tufts were formed at the junction of the paired colonies, two paired isolates were estimated to be sexually compatible (Tables 2 and 3). Such sexual compatibility tests were utilized to designate a provisional mating type code utilizing the letter “M” for each SBI. SBIs obtained from each of the field isolates 189, Rh28 and 1R4 of AG-1 IC were assigned the letters “a”, “b” and “c” (*i.e.*, Ma1, Ma2, Mb1, Mb2, etc.), respectively, while the field isolates SA-1, H10-28 and H10-268 of AG-2-2 IV were assigned the letters “x”, “y” and “z”, respectively.

### Microscopic hyphal anastomosis reactions

All field isolates of the two AGs and their SBI progenies were tested for hyphal anastomosis reactions. Isolates were grown on a 9 cm Petri dish containing 20 mL PDA for 3 d at 25°C in the dark. A small mycelial plug of each isolate was taken from the advancing margin, paired 3–4 cm apart on a glass slide and thinly covered with water agar (WA) medium. Glass slides with an agar plug were placed on a plastic box (100×200×30 mm) with a wet sterile paper towel and incubated for 2 d at 25°C in the dark. Each pairing was replicated three times. The overlapping hyphae of two grown colonies were stained with 0.05% cotton blue dissolved in 50% acetic acid to observe hyphal fusion at 100× magnification. Hyphal anastomosis reactions (C2 and C3 reactions) were determined based on the categorization of Carling (9). Paired isolates showing no death of interacting cells at five contact points were estimated as somatically compatible (C3 reaction) and assigned as “C3”. Paired isolates showing cell death at contact points were estimated as somatically incompatible (C2 reaction) and assigned as “C2”. Paired isolates showing each C2 and C3 reaction at different contact points were estimated as somatically incompatible/compatible (C2/3 reaction) and assigned as “C2/3” (Tables 2 and 3).

### Isolation of putative heterokaryons

Hyphae from each tuft (putative heterokaryon) were picked up with sterilized forceps and transferred to Petri dishes containing 10 mL AWA. Hyphal tips which appeared from the tuft were cut and transferred on PDA plates and incubated at 25°C in the dark. The colonies formed from these hyphae were saved as tuft isolates. Hyphal-tipped isolates from tufts were denoted by the letter “T” and a number relating to the tuft, *e.g.*, T5, and usually followed by a reference to the SBIs, which gave rise to the tuft, *e.g.*, T5(3*×4*). Tuft isolates formed between heterothallic SBIs obtained from the same parental field isolates were assigned as intra-B×B tuft isolates, and from different field isolates as inter-B×B tuft isolates (Hom-Hom pairings). Tuft isolates formed between heterothallic SBIs and their parental field isolates were assigned as intra-F×B tuft isolates, and between SBIs and their non-parental (different) field isolates as inter-F×B tuft isolates (Het-Hom pairings). Various combinations for tuft formation in this experiment are shown in Table S1. Characteristics of the tufts resulting from pairings of various types were examined and described.

### Determination of SCGs

Somatic compatibility reactions were performed using PDA medium. Five small disks from PDA plugs of each field isolate or tuft isolate of *T. cucumeris* AG-1 IC and -2-2 IV were placed about 2 cm apart in PDA Petri dishes and incubated at 25°C for 5–14 d. Isolates within an AG that grew together and failed to show a demarcation line at the colony junction were classified under the same SCG, whereas isolates within an AG exhibiting a demarcation line were classified into different SCGs (Fig. S1A). Tests were repeated 3 times each with two replicate plates.

### Heterokaryotic vigor test

Heterokaryotic vigor (heterosis) of tuft isolates was evaluated by the hyphal growth rate and pathogenicity.

#### Growth

The field isolates Rh28 and 1R4 of AG-1 IC, and their respective SBIs and tuft isolates were incubated at 25°C to compare hyphal growth among related isolates. The procedure is described as follows: All test isolates were grown on PDA for 3 days. The incubation temperature was set at 25°C. A single plug of 5 mm diameter of each isolate was cut from the edge of the hyphal colony, transferred to the margin of a 9 cm Petri dish containing 10 mL PDA, and incubated at the corresponding incubation temperature in the dark. Three radial lines were drawn from the center of the PDA plug on the back of each Petri dish. The hyphal growth rate is expressed as the increase in the radial colony measured every 24 h until the hyphae reached the fringe of the Petri dish, and calculated on the basis of three replicates.

#### Pathogenicity

The emergence rate of radish (*Raphanus sativus* L.) seeds was used to measure the pathogenicity of field isolates Rh28 and 1R4, and their respective SBIs and tuft isolates. Briefly, a 15 mm^2^ PDA agar plug with actively growing mycelia of each of the tested isolates was cut from the edge of a hyphal colony and placed on a 9 cm Petri dish containing 10 mL of 2% WA. After 3 days’ incubation at 25°C, 5 g sterilized soil was spread over the Petri dish. Radish seeds were surface disinfected by soaking in 10% sodium hypochlo-rite for 10 min under a vacuum, and then rinsed with sterilized water three times. Fifteen radish seeds about 10 mm apart from each other were seeded on each Petri dish, covered with additional 5 g sterilized soil, and watered with 10 mL sterilized water. After 48 h incubation at 30°C in the dark, the Petri dishes were placed in a growth chamber (14 light/10 dark h^−1^) at 25°C for 1-week incubation. Disease severity was estimated by calculating the number of germinated seedlings surviving at the 9 d after seeding. All isolates were tested four times with three replications per trial.

### DNA extraction and AFLP analysis

AFLP analysis was performed using extracted genomic DNA. DNA was extracted from field isolates, SBIs and tuft isolates using the method reported by Yoder (50) with modifications. Three small agar plugs of actively growing mycelium on PDA of each isolate were removed from the growing margin of 3-day-old cultures and transferred to a 9 cm diameter Petri dish containing 10 mL potato dextrose broth (PDB). After 5–6 days of incubation at 25°C in the dark, mycelial mats were collected by filtration onto filter paper, washed with sterile distilled water, blotted dry and frozen at −80°C until use. The DNA from each mycelium was extracted using the methodology described by Toda and Hyakumachi (41). The genomic DNA was diluted to 10 ng μL^−1^, following quantification of DNA using a spectrophotometer (Amersham Biotech, Piscataway, NJ). The principle of AFLP analysis is described in detail by Vos *et al.* (43). DNA digestion and ligation reactions for AFLP were performed according to the manual supplied with the AFLP kit (Applied Biosystems, Foster City, CA). Select primer combination (*Eco* RI-AG/*Mse* I-CA) was used for selective amplifications. Eight microliters of AFLP products were subjected to electrophoresis in a 15% polyacrylamide gel. The gel was stained serially with 0.1% AgNO_3_ and 1.5% NaOH containing formaldehyde for 10 min. The AFLP profile of fingerprints was visualized with a transilluminator and visible light. The reproducibility of the AFLP analysis results was tested by repeating the entire AFLP procedure three times using the same genomic DNA.

### Statistical analysis

Experiments of hyphal growth rates and pathogenicity were conducted three times and data from the repeated trials were pooled. Data were subjected to analysis of variance (ANOVA) and treatment means were compared by Fisher’s least significant difference (LSD) test.

## Results

### Heterokaryon formation between SBIs or between SBIs and their parental or non-parental field isolates

Different characteristics of tufts formed between intra-B×B, intra-F×B, inter-B×B and inter-F×B (Fig. S1) were observed. Fibrous tufts were formed between SBIs obtained from parental and non-parental field isolates (intra-B×B and inter-B×B) of *T. cucumeris* AG-1 IC and AG-2-2 IV, whereas sparse fibrous tuft or compact tufts were formed between SBIs and their parental or non-parental field isolates (intra-F×B and inter-F×B) of *T. cucumeris* AG-1 IC (Figs. S1B and S1C). Only compact tufts were formed between SBIs and their parental or non-parental field isolates (intra-F×B and inter-F×B) of AG-2-2 IV (Fig. S1D). Reactions of all tuft types are presented in Table S2. Representative tuft isolates produced by Hom-Hom and Het-Hom pairings among isolates of a common parent (intra-B×B and intra-F×B) and of different parents (inter-B×B and inter-F×B) with their somatic compatibility groupings are listed in Tables 4 and 5, respectively.

### Somatic compatibility reactions between field isolates and intra-B×B tuft isolates within *T. cucumeris* AG-1 IC, and within AG-2-2 IV

Letters “a”, “b” and “c” were assigned to distinguish mating types of SBIs obtained from each of the three field isolates 189, Rh28 and 1R4 of AG-1 IC, *i.e.*, the mating type of SBIs obtained from the field isolate 189 was assigned with “SBIs-Ma”. Similarly, field isolates SA-1, H10-28 and H10-268 of AG-2-2 IV were assigned the letters “x”, “y” and “z”, respectively. Each SBI was assigned an Arabic number and an asterisk, *i.e.*, 1*, 2*, 3* and so on. Nine SBIs (1*, 2*, 3*, 4*, 5*, 6*, 7*, 8* and 9*) obtained from the field isolate 189 of *T. cucumeris* AG-1 IC were tested for tuft formation. Five SBIs (1*, 6*, 7*, 8* and 9*) formed tufts with the other four SBIs (2*, 3*, 4* and 5*), but did not form any tuft among themselves. Those four SBIs (2*, 3*, 4* and 5*) also did not form tufts among themselves. These nine SBIs were divided into two mating types depending on the tuft formation results. Five SBIs (1*, 6*, 7*, 8* and 9*) were grouped into mating type 1, assigned -Ma1, while the other four SBIs (2*, 3*, 4* and 5*) were assigned as mating type 2 (-Ma2). Thirteen tuft isolates were formed between SBIs-Ma1 and -Ma2. The 13 tuft isolates were found to belong to three SCGs, based on the characteristics of the incompatibility reactions. Only one tuft isolate T5-[2*×7*] was classified under the same SCG as the parental field isolate 189, and labeled as SCG-1. Two tuft isolates, T2-[1*×4*] and T11-[4*×7*], were grouped as SCG-2, whereas the other ten tuft isolates (T1-[1*×3*], T3-[1*×5*], T4-[2*×6*], T6-[2*×8*], T7-[2*×9*], T8-[3*×6*], T9-[3*×7*], T10-[3*×8*], T12-[4*×8*] and T13-[5*×8*]) were classified as SCG-3. Except for SCG-1, to which parental field isolate 189 belonged, the other two SCGs (SCG-2 and SCG-3) were new SCGs (Table 4). The frequency of the occurrence of new SCGs among these 13 tuft isolates was 15.4% (Table 6). Seven SBIs (2*, 7*, 11*, 15*, 23*, 35*, 79*) obtained from field isolate SA-1 of *T. cucumeris* AG-2-2 IV were tested for tuft formation and produced nine tuft isolates. All nine tuft isolates formed between SBIs-Mx1 (15*, 35* and 79*) and -Mx2 (2*, 7*, 11* and 23*) showed somatic incompatibility with the parental field isolate SA-1, and were divided into three new SCGs: SCG-2 (T3-[2*×79*]), SCG-3 (T5-[7*×79*]) and SCG-4 (T1-[2*×15*], T2-[2*×35*], T4-[7*×35*], T6-[11*×35*], T7-[23*×35*], T8-[23*×79*] and T9-[11*×79*]) (Table 4). The frequency of the occurrence of new SCGs among these nine tuft isolates was 33.3% (Table 7). Similar results were obtained from *T. cucumeris* AG-1 IC isolate 1R4, and AG-2-2 IV isolates H10-28 and H10-268. Eleven, 16 and 14 tuft isolates were produced from IR4, H10-28 and H10-268, with 27.3%, 12.5% and 21.4% occurrence of new SCGs, respectively (Tables 6 and 7). The SCG groupings of these tuft isolates are presented in supplementary table 3 (Table S3).

### Somatic compatibility reactions among field isolates and intra-FxB tuft isolates within *T. cucumeris* AG-1 IC, and within AG-2-2 IV

The SBIs produced within each isolate were tested for tuft formation with their parent isolate and the resulting tuft isolates were sorted into SCGs based on the characteristics of their incompatibility reactions, as performed previously. Nine tuft isolates formed between SBIs-Mb1/2 (-Mb1: 1*, 3*, 4*, 5* and 9*; -Mb2: 6*, 7*, 8* and 10*) and their parental field isolate Rh28 were divided into two SCGs. Tuft isolates T1-[Rh28-F×1*] and T5-[Rh28-F×6*] were grouped as SCG-1, similar to parental field isolate Rh28. The other seven tuft isolates (T2-[Rh28-F×3*], T3-[Rh28-F×4*], T4-[Rh28-F×5*], T6-[Rh28-F×7*], T7-[Rh28-F×8*], T8-[Rh28-F×9*], T9-[Rh28-F×10*]) were sorted under a new SCG-2 (Table 4). The frequency of the occurrence of new SCGs among these nine tuft isolates was 11.1% (Table 6). All eight tuft isolates formed between SBIs-My1/2 (My1: 7*, 13*, 25* and 27*; My2: 30*, 32*, 37* and 39*) and their parental field isolate H10-28 showed somatic incompatibility with the parental field isolate H10-28, and were divided into three new SCGs: SCG-2 (T1-[H10-28-F×7*]), SCG-3 (T6-[H10-28-F×32*]) and SCG-4 (T2-[H10-28-F×13*], T3-[H10-28-F×25*], T4-[H10-28-F×27*], T5-[H10-28-F×30*], T7-[H10-28-F×37*], T8-[H10-28-F×39*]) (Table 4). The frequency of the occurrence of new SCGs among these eight tuft isolates was 37.5% (Table 7). The results obtained in pairings between the SBIs and their parent field isolates within *T. cucumeris* AG-1 IC, and within AG-2-2 IV are shown in Tables 6 and 7 and in supplementary table 3 (Table S3).

### Somatic compatibility reactions among field isolates and inter-BxB tuft isolates within *T. cucumeris* AG-1 IC, and within AG-2-2 IV

All tuft isolates formed between SBIs from different parental field isolates showed somatic incompatibility with their parental and non-parental field isolates. Eleven tuft isolates formed between SBIs-Mb1/2 (-Mb1: 1* and 3*; -Mb2: 2* and 16*) of Rh28 and SBIs-Mc1/2 (-Mc1: 24* and 25*; -Mc2: 6*, 8* and 13*) of 1R4 were divided into nine new SCGs. Parental field isolates Rh28 and 1R4 were grouped as SCG-1 and SCG-2, respectively. SCG-3 (T1-[Rh28-1*×1R4-8*]), SCG-4 (T2-[Rh28-1*×1R4-13*]), SCG-5 (T3-[Rh28-1*×1R4-24*]), SCG-6 (T4-[Rh28-2*×1R4-8*]), SCG-7 (T5-[Rh28-2*×1R4-24*], T6-[Rh28-2*×1R4-25*] and T10-[Rh28-7*×1R4-8*]), SCG-8 (T7-[Rh28-3*×1R4-6*]), SCG-9 (T8-[Rh28-3*×1R4-13*]), SCG-10 (T9-[Rh28-3*×1R4-24*]) and SCG-11 (T11-[Rh28-16*×1R4-24*]) were new SCGs (Table 5). The frequency of the occurrence of new SCGs among these 11 tuft isolates was 81.8% (Table 6). Eleven tuft isolates formed between SBIs-Mz1/2 (-Mz1: 1* and 4*; -Mz2: 2* and 3*) of H10-268 and SBIs-My1/2 (-My1: 7* and 13*; -My2: 30* and 32*) of H10-28 were divided into eight new SCGs. Parental field isolates H10-268 and H10-28 were grouped as SCG-1 and SCG-2, respectively. SCG-3 (T1-[H10-268-1*×H10-28-7*]), SCG-4 (T2-[H10-268-1*×H10-28-13*], T7-[H10-268-4*×H10-28-13*] and T9-[H10-268-3*×H10-28-13*]), SCG-5 (T3-[H10-268-2*×H10-28-7*], SCG-6 (T4-[H10-268-2*×H10-28-13*]), SCG-7 (T5-[H10-268-2*×H10-28-32*]), SCG-8 (T6-[H10-268-4*×H10-28-7*] and T8-[H10-268-3*×H10-28-7*]) SCG-9 (T10-[H10-268-3*×H10-28-30*]), SCG-10 (T11-[H10-268-3*×H10-28-32*]) were new SCGs (Table 5). The frequency of the occurrence of new SCGs among these 11 tuft isolates was 72.7% (Table 7). The additional results obtained from pairings of the SBIs of the two isolates within *T. cucumeris* AG-1 IC and within AG-2-2 IV are listed in Tables 6 and 7 and in supplementary Table 4 (Table S4).

### Somatic compatibility reactions among field isolates and inter-FxB within *T. cucumeris* AG-1 IC, and within AG-2-2 IV

All five tuft isolates formed between SBIs-Mc1/2 (-Mc1: 1*, 24* and 25*; -Mc2: 8* and 13*) of *T. cucumeris* AG-1 IC 1R4 and field isolate 189 showed somatic incompatibility with their parental isolates 189 (SCG-1) and 1R4 (SCG-2), and were divided into five new SCGs: SCG-3 (T1-[189-F×1R4-1*]), SCG-4 (T2-[189-F×1R4-8*]), SCG-5 (T3-[189-F×1R4-13*]), SCG-6 (T4-[189-F×1R4-24*]) and SCG-7 (T5-[189-F×1R4-25*]) (Table 5). The frequency of the occurrence of new SCGs among these five tuft isolates was 100% (Table 6). All six tuft isolates formed between SBIs-Mz1/2 (-Mz1: 1*, 4* and 8*; -Mz2: 2*, 3* and 7*) of *T. cucumeris* AG-2-2 IV H10-268 and field isolate H10-28 showed somatic incompatibility with their parental field isolates H10-28 (SCG-1) and H10-268 (SCG-2), and were divided into six new SCGs: SCG-3 (T1-[H10-28-F×H10-268-1*]), SCG-4 (T2-[H10-28-F×H10-268-2*]), SCG-5 (T3-[H10-28-F×H10-268-3*]), SCG-6 (T4-[H10-28-F×H10-268-4*]), SCG-7 (T5-[H10-28-F×H10-268-7*]) and SCG-8 (T6-[H10-28-F×H10-268-8*]) (Table 5). The frequency of the occurrence of new SCGs among these six tuft isolates was 100% (Table 7). The additional results obtained from pairings of the SBIs of an isolate with its non-parental field isolate within *T. cucumeris* AG-1 IC and within AG-2-2 IV are listed in Tables 6 and 7 and in supplementary Table 4 (Table S4).

### Variation in hyphal growth of *T. cucumeris* AG-1 IC

**Fig. 1:**
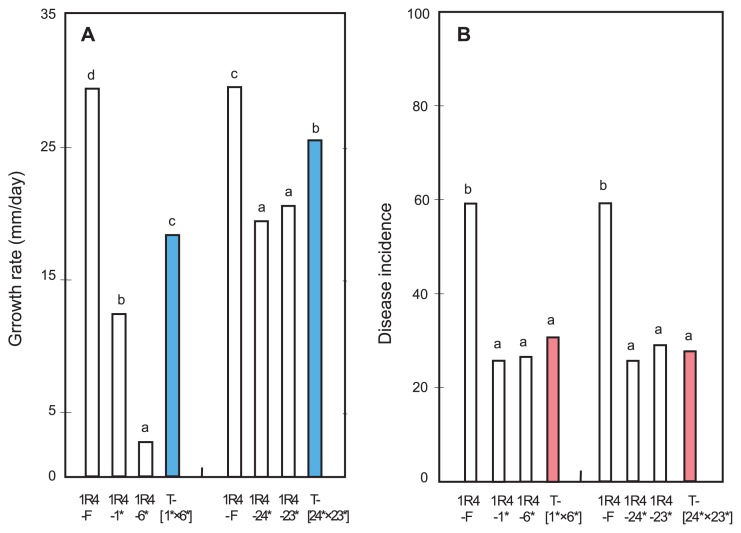
Growth rate and disease incidence of *T. cucumeris* AG-1 IC field isolate 1R4, its SBIs and heterokaryotic intra-B×B tuft isolates. **A**, Growth rate at 28°C. **B**, Disease incidence. “F” indicates field isolate 1R4. Asterisk indicates SBI obtained from 1R4. “T” indicates tuft isolate formed between SBI-Mc1 and -Mc2 of 1R4. Values with the same letters are not significantly different according to Fisher’s LSD test at *P*=0.05.

**Fig. 2:**
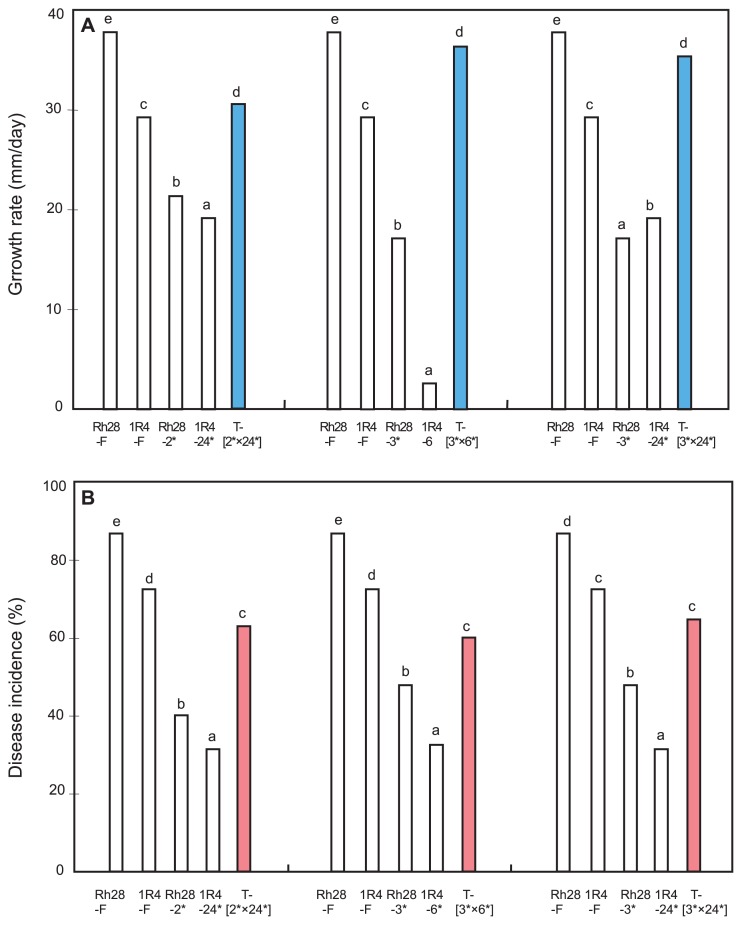
Growth rate and disease incidence of *T. cucumeris* AG-1 IC field isolates 1R4 and Rh28, their SBIs and heterokaryotic inter-B×B tuft isolates. **A**, Growth rate at 28°C. **B**, Disease incidence. “F” indicates field isolate Rh28 or 1R4. Asterisk indicates SBI obtained from Rh28 or 1R4. “T” indicates tuft isolate formed between Rh28-SBIs and 1R4-SBIs. Values with the same letters are not significantly different according to Fisher’s LSD test at *P*=0.05.

In general, the majority of tuft isolates showed faster hyphal growth than their contributing SBIs. For example, four out of five intra-B×B tuft isolates formed between SBIs-Mc1 and -Mc2 from 1R4 grew faster than both contributing -Mc1/-Mc2 SBIs, although their growth rates did not exceed the rate of their parental isolate 1R4. A remnant tuft isolate [12*×20*] grew faster than one of its contributing SBI 20* but slower than SBI 12* (Figs. 1A and S2). Inter-B×B tuft isolates formed between Rh28-SBIs and 1R4-SBIs all grew faster than both of their contributing Rh28-SBIs and 1R4-SBIs. They also grew faster than parental isolate 1R4, but not compared to parental isolate Rh28 (Fig. 2A). Inter-F×B tuft isolates formed between field isolate Rh28 (Rh28-F) and 1R4-SBIs showed a faster growth rate than their contributing 1R4-SBIs, but their growth rates did not exceed that of field isolate Rh28. Inter-F×B tuft isolates formed between field isolates 1R4 (1R4-F) and Rh28-SBIs grew faster than their contributing Rh28-SBIs; some tuft isolates such as [1R4-F×Rh28-1*] and [1R4-F×Rh28-2*] grew faster than field isolate 1R4 (Fig. S3).

### Variation in pathogenicity of *T. cucumeris* AG-1 IC

Field isolate Rh28 was more pathogenic than field isolate 1R4. Almost all SBIs from 1R4 showed substantially reduced pathogenicity, while the intra-B×B tuft isolates formed between SBIs-Mc1 and -Mc2 from 1R4 showed similar pathogenicity to their contributing -Mc1/-Mc2 SBIs. (Figs. 1B and S4). The pathogenicity of SBIs from Rh28 was more varied than SBIs from 1R4. Although almost all intra-B×B tuft isolates formed between SBIs-Mb1 and -Mb2 showed increased pathogenicity compared to their contributing -Mb1/-Mb2 SBIs, a few tuft isolates such as isolate [1*×2*] showed higher pathogenicity than their parental isolate Rh28 (Fig. S5). All of the Inter-B×B tuft isolates formed between Rh28-SBIs and 1R4-SBIs were more pathogenic than both of their contributing Rh28-SBIs and 1R4-SBIs (Fig. 2B).

### AFLP analysis

**Fig. 3:**
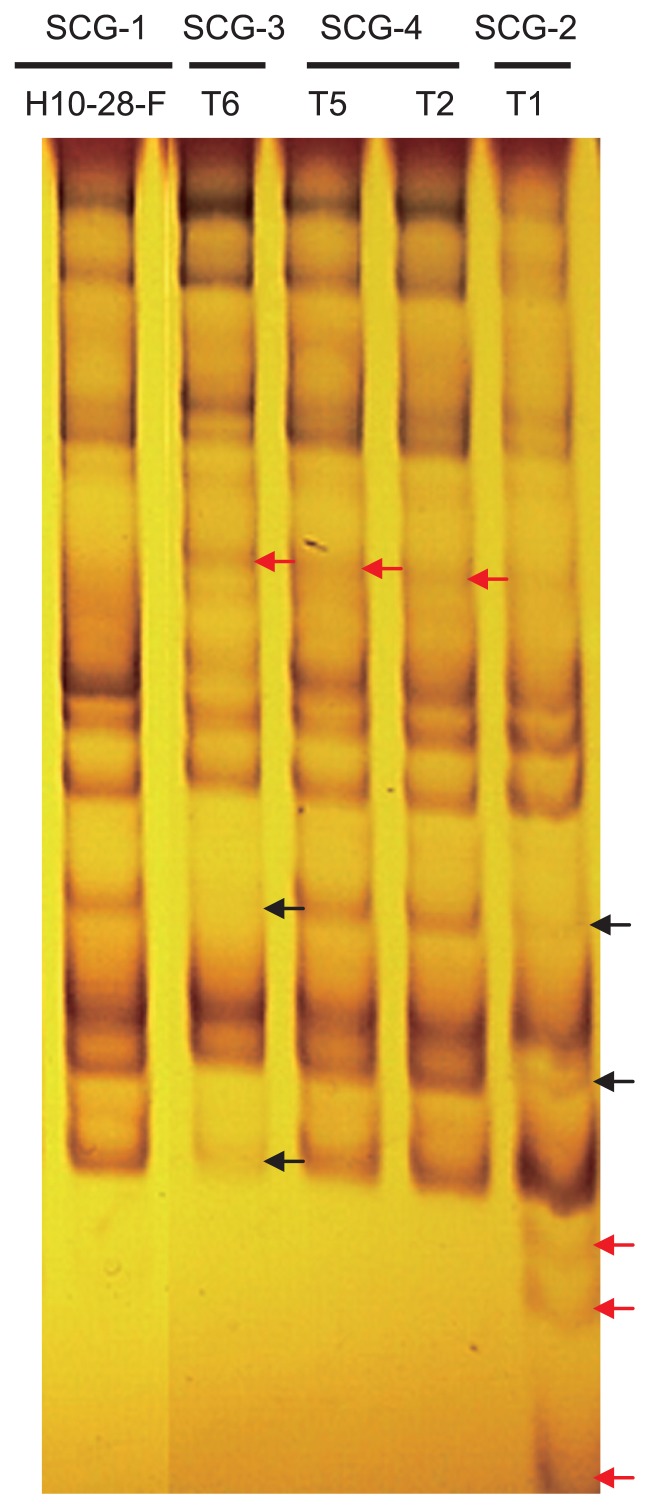
AFLP profile of the fingerprints of *T. cucumeris* AG-2-2 IV field isolate H10-28 and intra-F×B tuft isolates formed between H10-28 and its SBIs. The primer pair used for selective amplifications was *Eco*RI-AG/*Mse*I-CA. Black arrows indicate the specific markers present only in field isolate H10-28 and lost in tuft isolates. Red arrows indicate the specific markers present only in the tuft isolates but not in field isolate H10-28.

Tuft isolates [Rh28-F×1R4-8*] and [1R4-F×Rh28-7*] had a common and specific profile of fingerprints of each of their contributing parental isolates. Tuft isolate [Rh28-F×1R4-8*] had a specific profile of five fingerprints but had lost two of Rh28-F; it contained three but had lost three of 1R4-24* (Fig. S6). Tuft isolate [1R4-F×Rh28-7*] had a specific profile of seven fingerprints but had lost three of 1R4-F; it contained two but had lost one of Rh28-7* (Fig. S7). The differences in the AFLP fingerprint profile showed that parental field isolate H10-28-F and its tuft isolates belonged to different SCGs (Fig. 3). Tuft isolate T1-[H10-28-F×7*] belonging to SCG-2 had a specific profile of three fingerprints that were absent in field isolate H10-28-F, and had lost two that were present in field isolate H10-28-F. Tuft isolate T6-[H10-28-F×32*] belonging to SCG-3 had a specific profile of one fingerprint that was absent in field isolate H10-28-F, and had lost two that were present in field isolate H10-28-F. Tuft isolates T2-[H10-28-F×13*] and T5-[H10-28-F×30*] belonging to SCG-4 had a specific profile of one fingerprint that was absent in field isolate H10-28-F (Fig. 3).

## Discussion

The population biology of many filamentous fungi has been studied using SCGs (3, 34, 35). Individual isolates that can fuse with each other without cell death are said to be somatically compatible and therefore these isolates belong to the same SCG. If isolates form a somatic incompatibility reaction (demarcation line) (Fig. S1A), these two tested isolates are not genetically identical and belong to different SCGs. Yang *et al.* (47) reported that some pairings of field isolates of *T. cucumeris* AG-8 yielded tuft formation but some did not. Julian *et al.* (24) showed that the somatic incompatibility reactions did not correlate with tuft formation in *T. cucumeris* AG-1 IC. In our study, tufts were stably and repeatedly formed between SBIs belonging to different mating groups within *T. cucumeris* AG-1 IC and within AG-2-2 IV, while somatic incompatibility or compatibility reactions among SBIs were not stable, and there was no relationship with tuft formation (36, 41), as Julian *et al.* (24) reported. No tuft formation was observed among field isolates from Het-Het pairings within AG-1 IC and within AG-2-2 IV, respectively (Table S2). All field isolates from AG-1 IC and AG-2-2 IV used in this study showed somatic incompatibility with each other (data not shown).

The somatic incompatibility reactions of *T. cucumeris* are not very well understood but are thought to be encoded in the nucleus (4). McCabe *et al.* (29) found that some hyphal tip subcultures generated from isolates of *T. cucumeris* AG-4 showed somatic incompatibility with their respective parental field isolates. Nuclear activity in hyphal tip cells or changes in nuclear or mitochondrial types or mutations are all possible mechanisms that might result in the above reported changes in somatic compatibility.

SCG diversities of *Fusarium graminearum* from both wheat and barley were genetically highly variable even within a very small area (7, 8, 30). Rosewich *et al.* (37) also reported that a high degree of gene flow and regular outcrossing occurs between populations within field populations in AG-1 IA. Many reports showed that field isolates from several AGs of *T. cucumeris* could produce the sexual stage in nature. Dispersed SBIs cause foliage diseases in many types of plants (18, 26, 31, 38, 42, 49). We considered that outcrossing could occur among SBIs, or between SBIs and field isolates in the field. This may be an important contributor to the variability of SCGs.

In our study, somatic incompatibility reactions were observed between several tuft isolates formed between SBIs from the same or different field isolates, and between SBIs and their parental or non-parental (different) field isolates within AG-1 IC and within AG-2-2 IV. New SCGs that were different from parental field isolates occurred following tuft formations (Tables 6 and 7). The frequency of the occurrence of new SCGs was 15.4–27.3% and 11.1–25.0%, respectively, in intra-tuft isolates formed between SBIs obtained from the same parental field isolates (intra-B×B) and between SBIs and their parental field isolates (intra-F×B) in *T. cucumeris* AG-1 IC (Table 6). Similar results of 12.5–33.3% and 20.0–37.5%, respectively, were observed in intra-B×B and intra-F×B of AG-2-2 IV (Table 7). It was interesting that intra-tuft isolates obtained from SBIs within the same parental field isolates formed a relatively low percentage of new SCGs. For example, 10 of 13 tuft isolates formed between SBIs from AG-1 IC 189 belonged to SCG-3 (Tables 4, 6, and S3). SCG-3 was the most common new SCG of these tuft isolates.

Compared to the intra-tuft isolates formed within the same parental field isolates, inter-tuft isolates that originated from different field isolates showed a high occurrence of new SCGs. For example, high frequencies of occurrence, such as 62.5–87.5% and 100%, respectively, were observed in inter-tuft isolates formed between SBIs from different field isolates (inter-B×B) and between SBIs and their non-parental (different) field isolates (inter-F×B) within *T. cucumeris* AG-1 IC (Table 6). Similarly, high frequencies of the occurrence of new SCGs of 72.7%–81.8% and 87.5–100%, respectively, occurred between isolates of inter-F×B and inter-B×B in AG-2-2 IV (Table 7). All the tuft isolates that originated from different field isolates were somatically incompatible with their contributing field isolates. Almost all of these tuft isolates yielded unique SCGs and no SCG appeared commonly or repeatedly. Only a few tuft isolates showed somatic compatibility with other tuft isolates (Tables S3 and S4). AFLP analysis proved that all tuft isolates were heterokaryotic (Figs. S6 and S7).

Ogoshi and Ui (33) reported that the same SCGs (clones) within *T. cucumeris* AG-1 IA, AG-2-2 IV and AG-3 varied over time in the same rice, sugar-beet and potato fields. Inagaki (20) also reported that the same SCGs of *T. cucumeris* AG-1 IA were rarely present in the same rice paddy field over time, and SCGs were differed greatly between two seasons (spring and autumn) within a year or between seasons in two consecutive years. Our results suggested that heter-okaryon (tuft) formation, especially inter-B×B (Hom-Hom pairings) or inter-F×B (Het-Hom pairings) tuft formation, is probably very important for causing SCG variation in nature. Inagaki (20) also reported the appearance of several preponderant SCGs of *T. cucumeris* AG-1 IA in a rice field, but such preponderant SCGs did not continuously exist in the same field. We consider that one of the main reasons for the appearance of the dominant SCGs is intra-B×B or intra-F×B tuft formation.

Jacobson *et al.* (21) reported that randomly amplified polymorphic DNA (RAPD) marker analysis is more reliable and provides a higher resolution of genotype distribution in natural populations of the ectomycorrhizal fungus *Suillus granulatus* than somatic incompatibility reactions. RFLP genotypes of isolates from the same SCGs are identical in AG-1 IA (37). Ceresini *et al.* (13) studied the genetic diversity of field populations of *T. cucumeris* AG-3 PT and AG-3 TB in North Carolina using somatic compatibility and amplified fragment length polymorphism (AFLP) criteria. Their results indicated that isolates of AG-3 TB are represented by fewer SCGs and AFLP profiles of fingerprints than isolates of AG-3 PT.

In our study, AFLP profiles of fingerprints differed among SCGs, such as among parental field isolates and tuft isolates formed between the field isolate and its SBIs. On the other hand, AFLP profiles of the fingerprints of tuft isolates belonging to the same SCG were identical. The AFLP profiles of fingerprints of tuft isolates from different SCGs were distinct, and were not identical to their somatically incompatible parental field isolates (Fig. 3).

Growth and pathogenicity varied among different field isolates. The majority of SBIs showed reduced growth and pathogenicity compared to their parental field isolates (Figs. 1, 2, S2, S3, S4, and S5). Previous studies reported the reduced hyphal growth of SBIs from a field isolate of AG-5 (19), and the pathogenicity of SBIs obtained from a field isolate from flax (AG-unknown) (15) and single-protoplast isolates (SPIs) of AG-8 (48).

In our study, almost all tuft isolates grew faster than their contributing SBIs (Figs. 1A, 2A, S2 and S3), which was similar to the result of heterokaryon isolates synthesized between SBIs from isolates of AG-5 (19). Garza-Chapa and Anderson (15) reported that the heterokaryons were more virulent than either contributing SBIs. The pathogenicity of tuft isolates varied depending on the field isolates. Tuft isolates formed between SBIs of field isolate 1R4 had lower pathogenicity than 1R4 but were similar to their contributing SBIs, while tuft isolates derived from SBIs of Rh28 showed similar pathogenicity to Rh28 but increased pathogenicity compared to their individual SBIs (Figs. 1B and 2B).

We consider that the genes concerned with growth and pathogenicity are independent of each other. Furthermore, as the existence of the more common SCGs is uncertain under field conditions, heterokaryosis may not be directly related with the occurrence of new SCGs in nature, although our results suggest that heterokaryosis is probably a major contributor.

## Supplementary Material



## Figures and Tables

**Table 1:** List of field and single-basidiospore isolates (SBIs) of *T. cucumeris* AG-1 IC and AG-2-2 IV used in this study1)

Anastomosis Group	Field isolate	Host plant	Geographic origin	Mating type	SBI number
AG-1 IC	189	Cauliflower	USA	Ma1	1*, 6*, 7*, 8* 9*
				Ma2	2*, 3*, 4*, 5*, 10*
	Rh28	Sugar beet	Hokkaido, Japan	Mb1	1*, 3*, 4*, 5*, 9*
				Mb2	2*, 6*, 7*, 8*, 10*, 16*
	IR4	Pine	Canada	Mc1	1*, 2*, 12*, 18*, 22*, 24*, 25*
				Mc2	6*, 7*, 8*, 10*, 13*, 20*, 23*
AG-2-2 IV	SA-1	Sugar beet	Hokkaido, Japan	Mx1	1*, 4*, 15*, 21*, 35*, 79*
				Mx2	2*, 7*, 11*, 23*
	H10-28	Sugar beet	Hokkaido, Japan	My1	7*, 13*, 25*, 27*
				My2	30*, 32*, 37*, 39*
	H10-268	Sugar beet	Hokkaido, Japan	Mz1	1*, 4*, 8*, 9*, 10*, 11*
				Mz2	2*, 3*, 7*, 12*, 15*

1) SBIs obtained from each of the field isolates 189, Rh28 and 1R4 of AG-1 IC were assigned the letters “a”, “b” and “c”, respectively; while field isolates SA-1, H10-28 and H10-268 of AG-2-2 IV were assigned the letters “x”, “y” and “z”, respectively. Moreover, the SBIs derived from each field isolate were further classified into mating types 1 or 2 (SBI Ma1, SBI Ma2, SBI Mb1, etc.).

**Table 2:** Frequency of new somatic compatibility groups (SCGs) in tuft isolates of *T. cucumeris* AG-1 IC

Isolate/SBI	Somatic compatibility		Mating compatibility		AFLP profile
		
	1*[M1]1)	2*[M2]1)	1*[M1]	2*[M2]	
Isolate Rh28	C22)	C22)	+3)	+3)	B4)
SBIs-Mb1					
1*	C3	C2	−	+	C
3*	C2/3	C2	−	+	E
4*	C2	C2	−	+	B
5*	nt5)	nt	−	+	A
SBIs-Mb2					
2*	C2	C3	+	−	D
6*	C2	C2	+	−	B
7*	C2	C2	+	−	C
8*	nt	nt	+	−	B

1) 1* and 2* were randomly selected basidiospore isolates used as mating type tester isolates for mating type 1 (M1) and mating type 2 (M2), respectively.

2) Somatic hyphal interaction based on the type of interaction characterized by Carling (9). C2 = C2 reaction (somatic incompatibility); C3 = C3 reaction (somatic compatibility); C2/3 = both C2 and C3 reactions were observed at different contact points in the same glass slide.

3) (−) mating incompatibility (no tuft formation); (+) mating compatibility (tuft formation).

4) Designated grouping of the SBI according to its AFLP fingerprint profile.

5) nt = not tested.

**Table 3:** Frequency of new somatic compatibility groups (SCGs) in tuft isolates of *T. cucumeris* AG-2-2 IV

Isolate/SBI	Somatic compatibility		Mating compatibility		AFLP profile
		
	4*[M1]1)	2*[M2]1)	4*[M1]	2*[M2]	
Isolate SA-1	C2/32)	C2/32)	+3)	+3)	A4)
SBIs-Mx1					
1*	C3	C2	−	+	B
4*	C3	C2/3	−	+	D
15*	C2	C2	−	+	G
21*	C2/3	C2	−	+	H
SBIs-Mx2					
2*	C2/3	C3	+	−	C
7*	C2	C2	+	−	E
11*	C2/3	C2/3	+	−	F
23*	C2/3	C2/3	+	−	I

1) 4* and 2* were randomly selected basidiospore isolates used as mating type tester isolates for mating type 1 (M1) and mating type 2 (M2), respectively.

2) Somatic hyphal interaction based on the type of interaction characterized by Carling (9). C2 = C2 reaction (somatic incompatibility); C3 = C3 reaction (somatic compatibility); C2/3 = both C2 and C3 reactions were observed at different contact points in the same glass slide.

3) (−) mating incompatibility (no tuft formation); (+) mating compatibility (tuft formation).

4) Designated grouping of the SBI according to its AFLP fingerprint profile.

**Table 4:** Representative tuft isolates produced by Hom-Hom and Het-Hom pairings among isolates of a common parent (intra-B×B and intra-F×B) within *T. cucumeris* AG-1 IC and AG-2-2 IV

Intra SBI × SBI^a^ (Hom-Hom)			Intra Parent isolate × SBI^b^ (Het-Hom)		
	
Anastomosis Group	Isolate	SCG group	Anastomosis Group	Isolate	SCG group
AG-1 1C	189-F, T5-[2*×7*]	SCG 1	AG-1 1C	Rh 28-F, T1-[Rh28-F×1*], T5-[Rh28-F×6*]	SCG 1
	T2-[1*×4*], T11-[4*×7*]	SCG 2		T2-[Rh28-F×3*], T3-[Rh28-F×4*], T4-[Rh28-F×5*], T6-[Rh28-F×7*], T7-[Rh28-F×8*], T8-[Rh28-F×9*], T9-[Rh28-F×10*]	SCG 2
	T1-[1*x3*], T3-[1*×5*], T4-[2*×6*], T6-[2*×8*], T7-[2*×9*], T8-[3*×6*], T9-[3*×7*], T10-[3*×8*], T12-[4*×8*], T13-[5*×8*])	SCG 3			
AG-2-2 IV	SA-1-F	SCG 1	AG-2-2 IV	H10-28-F	SCG 1
	T3-[2*×79*]	SCG 2		T1-[H10-28-F×7*]	SCG 2
	T5-[7*×79*]	SCG 3		T6-[H10-28-F×32*]	SCG 3
	T1-[2*×15*], T2-[2*×35*], T4-[7*×35*], T6-[11*×35*], T7-[23*×35*], T8-[23*×79*], T9-[11*×79*]	SCG 4		T2-[H10-28-F×13*], T3-[H10-28-F×25*], T4-[H10-28-F×27*], T5-[H10-28-F×30*], T7-[H10-28-F×37*], T8-[H10-28-F×39*]	SCG 4

a Same as Intra-B×B = tuft isolates formed between SBI progenies from the same field isolate.

b Same as Intra-F×B = tuft isolates formed between a field isolate and its SBI progenies.

**Table 5:** Representative tuft isolates produced by Hom-Hom and Het-Hom pairings among isolates of different parents (inter-B×B and inter-F×B) within *T. cucumeris* AG-1 IC and AG-2-2 IV

Inter SBI × SBI^a^ (Hom-Hom)			Inter Parent isolate × SBI^b^ (Het-Hom)		
	
Anastomosis Group	Isolate	SCG group	Anastomosis Group	Isolate	SCG group
AG-1 1C	Rh 28-F	SCG 1	AG-1 1C	189-F	SCG 1
	IR4-F	SCG 2		IR4-F	SCG 2
	T1-[Rh28-1*×1R4-8*]	SCG 3		T1-[189-F×1R4-1*]	SCG 3
	T2-[Rh28-1*×1R4-13*]	SCG 4		T2-[189-F×1R4-8*]	SCG 4
	T3-[Rh28-1*×1R4-24*]	SCG 5		T3-[189-F×1R4-13*]	SCG 5
	T4-[Rh28-2*×1R4-8*]	SCG 6		T4-[189-F×1R4-24*]	SCG 6
	T5-[Rh28-2*×1R4-24*], T6-[Rh28-2*×1R4-25*], T10-[Rh28-7*×IR4-8*]	SCG 7		T5-[189-F×1R4-25*]	SCG 7
	T7-[Rh28-3*×1R4-6*]	SCG 8			
	T8-[Rh28-3*×1R4-13*]	SCG 9			
	T9-[Rh28-3*×1R4-24*]	SCG 10			
	T11-[Rh28-16*×1R4-24*]	SCG 11			
AG-2-2 IV	H10-268-F	SCG 1	AG-2-2 IV	H10-28-F	SCG 1
	H10-28-F	SCG 2		H10-268-F	SCG 2
	T1-[H10-268-1*×H10-28-7*]	SCG 3		T1-[H10-28-F×H10-268-1*]	SCG 3
	T2-[H10-268-1*×H10-28-13*], T7-[H10-268×H10-28-13*], T9-[H10-268-3*×H10-28-13*]	SCG 4		T2-[H10-28-F×H10-268-2*]	SCG 4
	T3-[H10-268-2*×H10-28-7*]	SCG 5		T3-[H10-28-F×H10-268-3*]	SCG 5
	T4-[H10-268-2*×H10-28-13*]	SCG 6		T4-[H10-28-F×H10-268-4*]	SCG 6
	T5-[H10-268-2*×H10-28-32*]	SCG 7		T5-[H10-28-F×H10-268-7*]	SCG 7
	T6-[H10-268-4*×H10-28-7*], T8-[H10-268-3*×H10-28-7*]	SCG 8		T6-[H10-28-F×H10-268-8*]	SCG 8
	T10-[H10-268-3*×H10-28-30*]	SCG 9			
	T11-[H10-268-3*×H10-28-32*]	SCG 10			

a Same as Inter-B×B = tuft isolates formed between SBI progenies from different field isolates.

b Same as Inter-F×B = tuft isolates formed between a field isolate and SBI progenies from another field isolate.

**Table 6:** Frequency of new somatic compatibility groups (SCGs) in tuft isolates of *T. cucumeris* AG-1 IC

	Intra-tuft isolate				Inter-tuft isolate					
		
	Intra-B×B^a^		Intra-F×B^b^		Inter-B×B^c^			Inter-F×B^d^		
Type of tuft isolate	189-B×B	1R4-B×B	Rh28-F×B	1R4-F×B	189-B× Rh28-B	189-B× 1R4-B	1R4-B× Rh28-B	189-F× 1R4-B	1R4-F× Rh28-B	1R4-F× 189-B
Number of tuft isolates	13	11	4	9	8	8	11	5	4	6
Number of SCGs	3	4	2	2	9	7	11	7	6	8
Number of new SCGs	2	3	1	1	7	5	9	5	4	6
Frequency of new SCGs	2/13 (15.4%)	3/11 (27.3%)	1/4 (25%)	1/9 (11.1%)	7/8 (87.5%)	5/8 (62.5%)	9/11 (81.8%)	5/5 (100%)	4/4 (100%)	6/6 (100%)

a Intra-B×B = tuft isolates formed between SBI progenies from the same field isolate.

b Intra-F×B = tuft isolates formed between a field isolate and its SBI progenies.

c Inter-B×B = tuft isolates formed between SBI progenies from different field isolates.

d Inter-F×B = tuft isolates formed between a field isolate and SBI progenies from another field isolate.

**Table 7:** Frequency of new somatic compatibility groups (SCGs) in tuft isolates of *T. cucumeris* AG-2-2 IV

Intra-tuft isolate							Inter-tuft isolate					
	
	Intra-B×B^a^			Intra-F×B^b^			Inter-B×B^c^			Inter-F×B^d^		
Type of tuft isolate	SA-1-B×B	H10-28-B×B	H10-268-B×B	SA-1-F×B	H10-28-F×B	H10-268-F×B	H10-268-B× H10-28-B	SA-1-B× H10-28-B	SA-1-B× H10-268-B	H10-28-F× H10-268-B	H10-268-F× H10-28-B	SA-1-F× H10-268-B
Number of tuft isolates	9	16	14	8	8	10	11	11	11	6	8	5
Number of SCGs	4	3	4	3	4	3	10	10	11	8	9	7
Number of new SCGs	3	2	3	2	3	2	8	8	9	6	7	5
Frequency of new SCGs	3/9 (33.3%)	2/16 (12.5%)	3/14 (21.4%)	2/8 (25%)	3/8 (37.5%)	2/10 (20%)	8/11 (72.7%)	8/11 (72.7%)	9/11 (81.8%)	6/6 (100%)	7/8 (87.5%)	5/5 (100%)

a Intra-B×B = tuft isolates formed between SBI progenies from the same field isolate.

b Intra-F×B = tuft isolates formed between a field isolate and its SBI progenies.

c Inter-B×B = tuft isolates formed between SBI progenies from different field isolates.

d Inter-F×B = tuft isolates formed between a field isolate and SBI progenies from another field isolate.

## References

[1] Adams GC, Sidhu GS (1988). *Thanatephorus cucumeris* (*Rhizoctonia solani*), a species complex of wide host range. Advances in Plant Pathology, Vol. 6, Genetics of Plant Pathogenic Fungi.

[2] Adams GC, Butler EE (1982). A re-interpretation of the sexuality of *Thanatephorus cucumeris* anastomosis group four. Mycologia.

[3] Adams GC, Hammar SA, Proffer T (1990). Vegetative compatibility in *Leucostoma persoonii*. Phytopathology.

[4] Adams GC, Sneh B, Jabaji-Hare S, Neate S, Dijst G (1996). Genetics of *Rhizoctonia solani* species. *Rhizoctonia* Species: Taxonomy, Molecular Biology, Ecology, Pathology and Disease Control.

[5] Anderson NA, Stretton HM, Groth JV, Flentje NT (1972). Genetics of heterokaryosis in *Thanatephorus cucumeris*. Phytopathology.

[6] Anderson NA (1982). The genetics and pathology of *Rhizoctonia solani*. Annu Rev Phytopathol.

[7] Bowden RL, Leslie JF (1992). Nitrate-nonutilizing mutants of *Gibberella zeae*(*Fusarium graminearum*) and their use in determining vegetative compatibility. Exp Mycol.

[8] Bowden RL, Leslie JF (1994). Diversity of *Gibberella zeae* at small spatial scales. Phytopathology.

[9] Carling DE, Sneh B, Jabaji-Hare S, Neate S, Dijst G (1996). Grouping in *Rhizoctonia solani* by hyphal anastomosis reaction. *Rhizoctonia* Species: Taxonomy, Molecular Biology, Ecology, Pathology and Disease Control.

[10] Carling DE, Baird RE, Gitaitis KA, Kuninaga S (2002). Characterization of AG-13, a newly reported anastomosis group of *Rhizoctonia solani*. Phytopathology.

[11] Carling DE, Pope EJ, Brainard KA, Carter DA (1999). Characterization of mycorrhizal isolates of *Rhizoctonia solani* from an orchid, including AG-12, a new anastomosis group. Phytopathology.

[12] Carling DE, Leiner RH, Kebler KM (1987). Characterization of a new anastomosis group (AG-9) of *Rhizoctonia solani*. Phytopathology.

[13] Ceresini PC, Shew HD, Vilgalys RJ, Rosewich UL, Cubeta MA (2002). Genetic structure of populations of *Rhizoctonia solani* AG-3 on potato in eastern North Carolina. Mycologia.

[14] Cubeta MA, Vilgalys R (1997). Population biology of the *Rhizoctonia solani* complex. Phytopathology.

[15] Garza-Chapa R, Anderson NA (1966). Behavior of single-basidiospore isolates and heterokaryons of *Rhizoctonia solani* from flax. Phytopathology.

[16] Glass NL, Jacobson DJ, Shiu PKT (2000). The genetics of hyphal fusion and vegetative incompatibility in filamentous ascomycete fungi. Annu Rev Genet.

[17] Hietala AM, Korhonen K, Sen R (2003). An unknown mechanism promotes somatic incompatibility in *Ceratobasidium bicorne*. Mycologia.

[18] Hsieh TF, Lin HF (1997). Distribution pattern of Chinese spinach leaf spots associated with basidiospore infection of *Thanatephorus cucumeris*. Plant Pathol.

[19] Homma Y, Ui T (1975). Cultural characters and growth rate of single-basidiospore isolates and synthesized heterokaryons of a field isolate of *Rhizoctonia solani* Kuhn. Memoirs Fac Agric Hokkaido Univ.

[20] Inagaki K (1998). Dispersal of rice sheath blight fungus, *Rhizoctonia solani* AG-1(IA), and subsequent disease development in paddy fields, from survey of vegetative compatibility groups. Mycoscience.

[21] Jacobson KM, Miller OK, Turner BJ (1993). Randomly amplified polymorphic DNA markers are superior to somatic incompatibility tests for discriminating genotypes in natural populations of the ectomycorrhizal fungus *Suillus granulatus*. Proc Nat Acad Sci USA.

[22] Johannesson H, Stenlid J (2004). Nuclear reassortment between vegetative mycelia in natural populations of the basidiomycete *Heterobasidion annosum*. Fungal Genet Biol.

[23] Julián MC, Debets F, Keijer J (1996). Independence of sexual and vegetative incompatibility mechanisms of *Thanatephorus cucumeris* (*Rhizoctonia solani*) anastomosis group 1. Phytopathology.

[24] Julian MC, Dullemans AM, Silfhout C, Keijer J (1997). Nuclear behavior in homokaryotic and heterokaryotic fruiting of *Thanatephorus cucumeris* (*Rhizoctonia solani*) anastomosis group 1, subgroup IC. Mycologia.

[25] Leslie JF (1993). Fungal vegetative compatibility. Annu Rev Phytopathol.

[26] Luke WJ, Pinckard JA, Wang SC (1974). Basidiospore infection of cotton bolls by *Thanatephorus cucumeris*. Phytopathology.

[27] MacNish GC, Carling DE, Sweetingham MW, Ogoshi A, Brainard KA (1995). Characterization of anastomosis group-10 (AG-10) of *Rhizoctonia solani*. Australasian Plant Pathol.

[28] May G (1988). Somatic incompatibility and individualism in the coprophilous basidiomycete, *Coprinus cinereus*. Trans Br Mycol Soc.

[29] McCabe PM, Gallacher MP, Deacon LW (1999). Evidence for segregation of somatic incompatibility during hyphal tip subculture of *Rhizoctonia solani* AG 4. Mycol Res.

[30] McCallum BD, Tekauz A, Gilbert J (2001). Vegetative compatibility among *Fusarium graminearum*(*Gibberella zeae*) isolates from barley spikes in southern Manitoba. Can J Plant Pathol.

[31] Naito S, Sneh B, Jabaji-Hare S, Neate S, Dijst G (1996). Basidiospore dispersal and survival. *Rhizoctonia* Species: Taxonomy, Molecular Biology, Ecology, Pathology and Disease Control.

[32] Ogoshi A (1972). On the perfect stage of anastomosis group 2 of *Rhizoctonia solani* Kühn. Ann. Phytopathological Soc Japan.

[33] Ogoshi A, Ui T (1983). Diversity of clones within an anastomosis group of *Rhizoctonia solani* Kühn in field of white potatoes, sugar beets, and rice plants. Ann. Phytopathological Soc Japan.

[34] Proffer TJ, Hart JH (1988). Vegetative compatibility groups in *Leucocytospora kunzei*. Phytopathology.

[35] Punja ZK, Grogan RG (1983). Hyphal interactions and antagonism among field isolates and single-basidiospore strains of *Athelia* (*Sclerotium*) *rolfsii*. Phytopathology.

[36] Qu P, Yamashita K, Toda T, Priyatmojo A, Kubota M, Hyakumachi M (2008). Heterokaryon formation in *Thanatephorus cucumeris*(*Rhizoctonia solani*) AG-1 IC. Mycol Res.

[37] Rosewich UL, Pettway RE, McDonald BA, Kistler HC (1999). High levels of gene flow and heterozygote excess characterize *Rhizoctonia solani* AG-1 IA (*Thanatephorus cucumeris*) from Texas. Fungal Genet Biol.

[38] Shew HD, Main CE (1985). Rhizoctonia leaf spot of flue-cured tobacco in North Carolina. Plant Dis.

[39] Sneh B, Burpee L, Ogoshi A (1991). Identification of *Rhizoctonia* species.

[40] Sneh B, Jabaji-Hare S, Neate S, Dijst G (1996). *Rhizoctonia* Species: Taxonomy, Molecular Biology, Ecology, Pathology and Disease Control.

[41] Toda T, Hyakumachi M (2006). Heterokaryon formation in *Thanatephorus cucumeris* anastomosis group 2-2 IV. Mycologia.

[42] Uchida JY, Aragaki M, Yahata PS (1986). Basidiospore formation by *Ceratobasidium* sp. on agar. Mycologia.

[43] Vos P, Hogers R, Bleeker M, Reijans M, van de Lee T, Frijters A, Pot J, Peleman J, Kuiper M, Zabeau M (1995). AFLP: A new technique for DNA finger printing. Nucleic Acids Res.

[44] Whitney HS, Parmeter JR (1963). Synthesis of heterokaryons in *Rhizoctonia solani* Kühn. Can J Bot.

[45] Worrall JJ (1997). Somatic incompatibility in basidiomycetes. Mycologia.

[46] Yang HA, Tommerup IC, Sivasithamparam K, O’Brien PA (1992). Heterokaryon formation with homokaryons derived from protoplasts of *Rhizoctonia solani* anastomosis group eight. Exp Mycol.

[47] Yang HA, Sivasithamparam K, O’Brien PA (1993). Mycelial interactions and the potential use of tuft formation in characterising *Rhizoctonia solani* isolates infecting cereals. Australian J Bot.

[48] Yang HA, Zhou J, Sivasithamparam K, O’Brien PA (1994). Variations in culture morphology and pathogenicity among protoplast-regenerated strains of *Rhizoctonia solani*. FEMS Microbiol Lett.

[49] Yik CP, Wong JL (1992). Characterisation of *Rhizoctonia solani* causing leaf rots of vegetables in Singapore. Singapore J Primary Ind.

[50] Yoder OC, Sidhu GS (1988). *Cochliobolus heterostrophus* cause of southern corn leaf blight. Advances in Plant Pathology, Vol. 6, Genetics of Plant Pathogenic Fungi.

