# Supplementary material for: Mechanism of the Generation of New Somatic Compatibility Groups within Thanatephorus cucumeris (Rhizoctonia solani)
Source: Microbes Environ. 2013 Aug 31;28(3):325–35. doi: 10.1264/jsme2.ME12214 (PMC4070963; doi:10.1264/jsme2.ME12214)
Supplement: Supplementary file 1 [file 28_325_s1.pdf]

Supplementary data

Mechanism of the Generation of New Somatic Compatibility Groups within *Thanatephorus cucumeris* (*Rhizoctonia solani*)

Ping Qu[1], Mary Grace B. Saldajeno[2], and Mitsuro Hyakumachi[2*]

[1]College of Life Sciences, Shaanxi Normal University, 199 South Chang'an Road, Xi'an 710062, China

[2]Laboratory of Plant Pathology, Faculty of Applied Biological Sciences, Gifu University, 1-1 Yanagido, Gifu 501-1193, Japan

*Corresponding author. E-mail: hyakumac@gifu-u.ac.jp

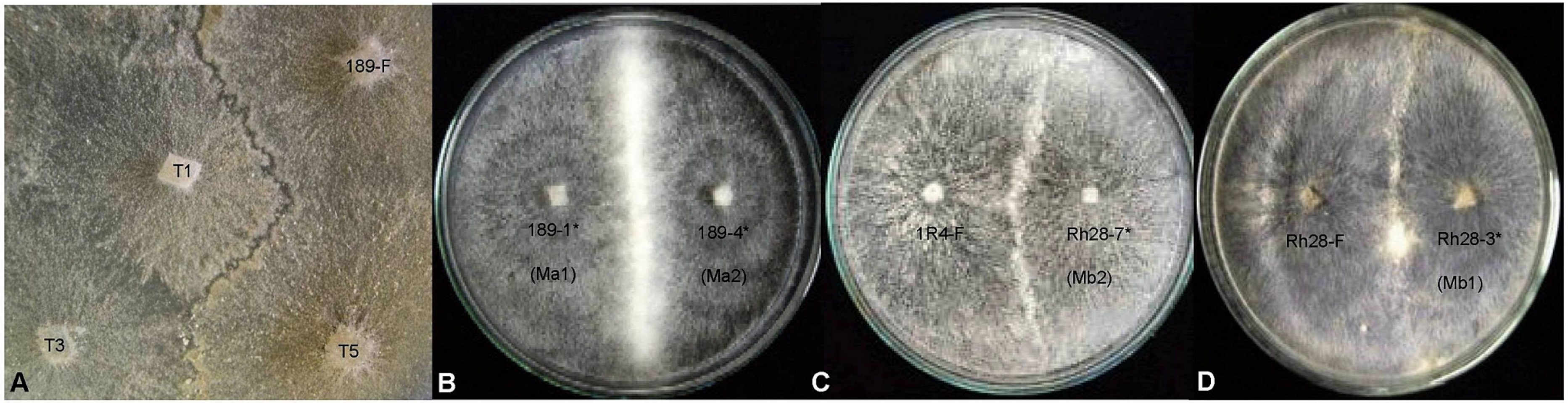

Figure S1. (A) Somatic compatibility reactions between *T. cucumeris* AG-1 IC parental field isolate 189 and its intra-B×B tuft isolates formed between SBIs-Ma1 and -Ma2. Somatic incompatibility reactions occurred between 189 and tuft isolate T1; between tuft isolates T1 and T5; and between T3 and T5. Field isolate 189 and tuft isolate T5 belong to the same somatic compatibility group (SCG-1), while tuft isolates T1 and T3 belong to SCG-3. (B) Fibrous tuft formed between SBIs-1* (Ma1) and 4* (Ma2) obtained from field isolate 189 of *T. cucumeris* AG-1 IC on PDCA culture with 0.5% charcoal concentration (intra-B×B tuft). (C) Sparse tuft formed between SBI-7*(Mb2) from field isolate Rh28 and its non-parental field isolate 1R4 of AG-1 IC (inter-F×B tuft). (D) Compact tuft formed between SBI-3*(Mb1) and its parental field isolate Rh28 of AG-1 IC (intra-F×B tuft).

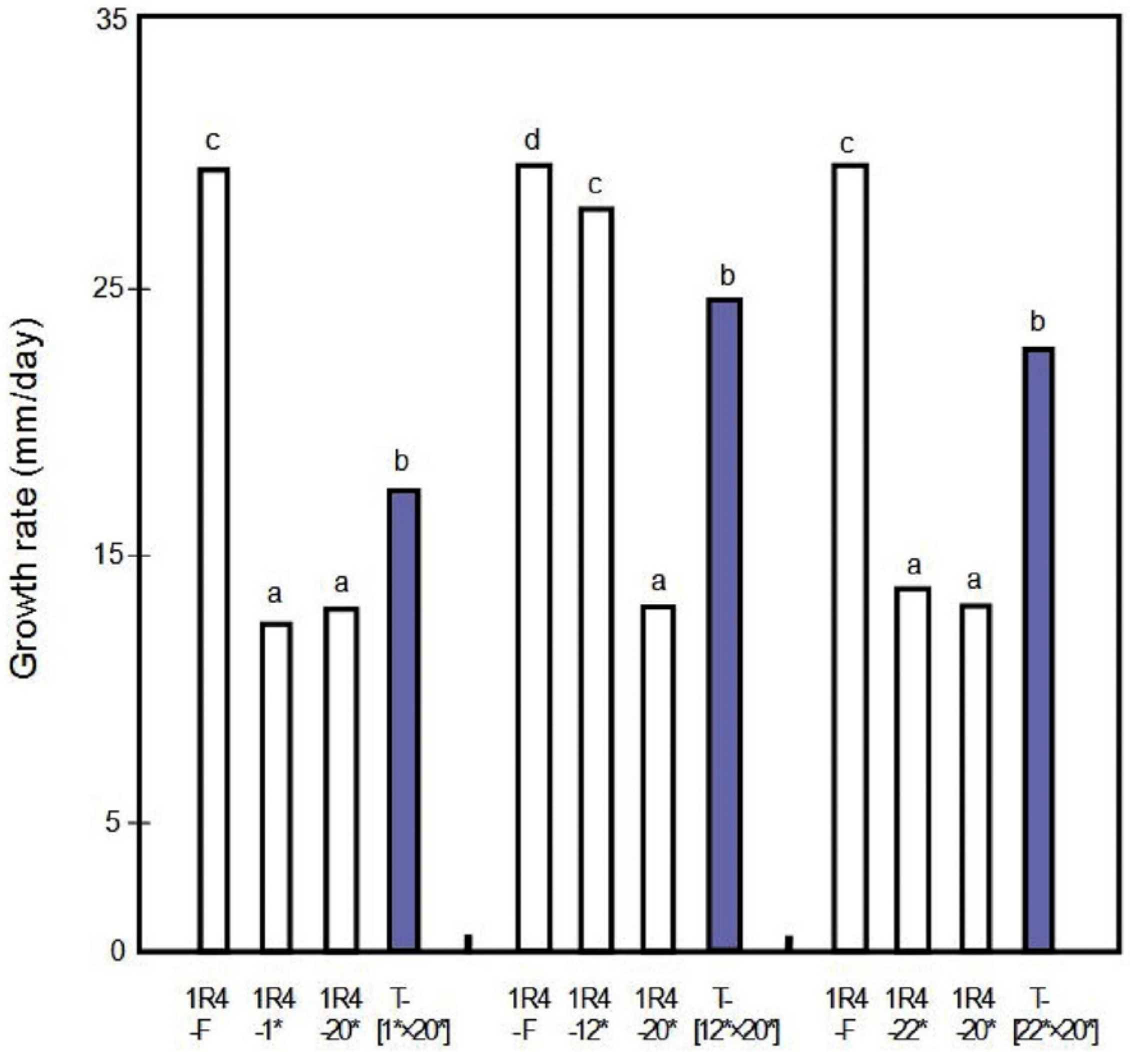

Figure S2. Growth rate of *T. cucumeris* AG-1 IC field isolate 1R4, its SBIs and heterokaryotic intra-B×B tuft isolates formed between SBIs-Mc1 and -Mc2 at 28°C. “F” indicates field isolate 1R4. Asterisk indicates SBI obtained from 1R4. “T” indicates tuft isolate formed between SBI-Mc1 and -Mc2 of 1R4. Values with the same letters are not significantly different according to Fisher’s LSD test at P=0.05.

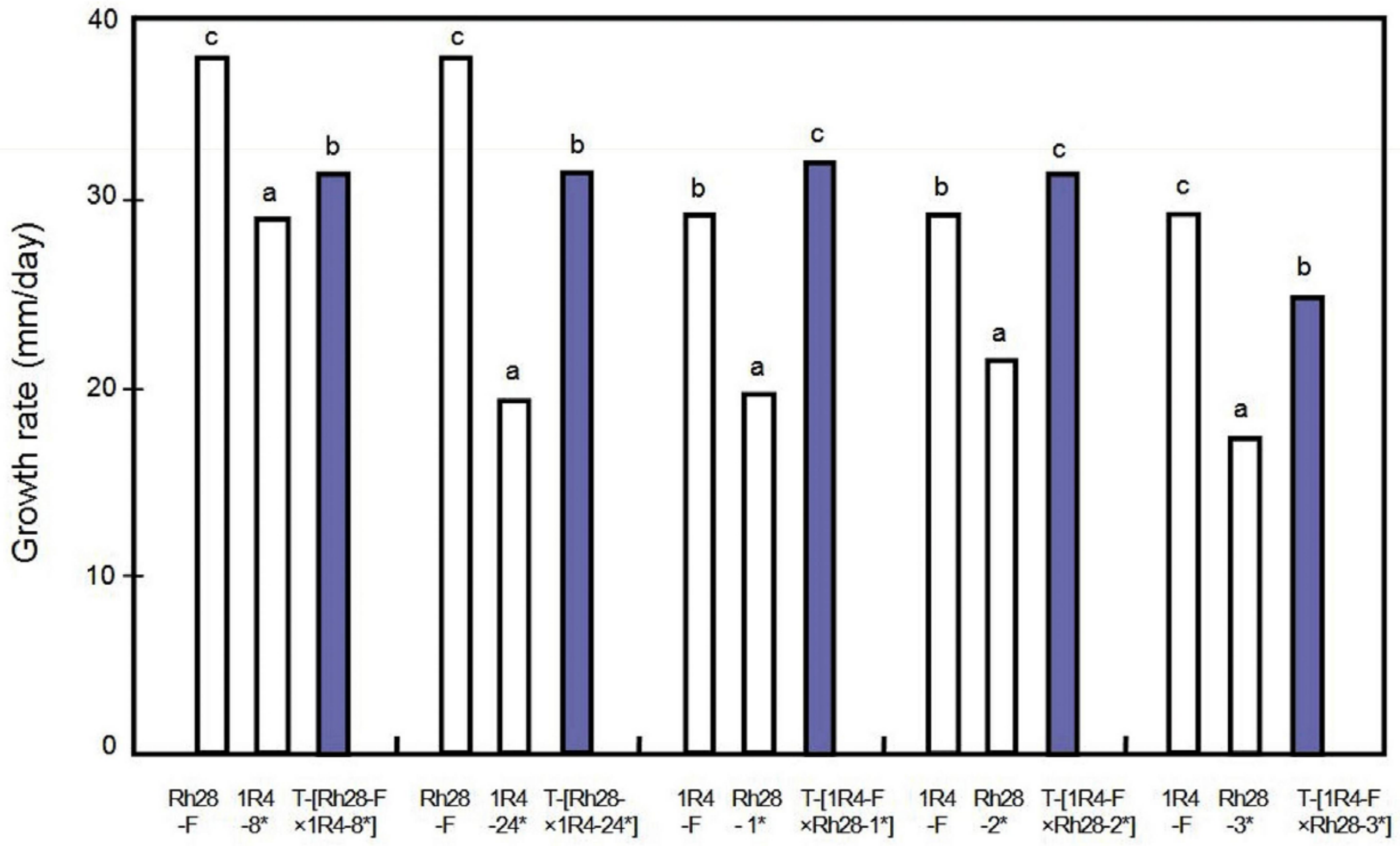

Figure S3. Growth rate of *T. cucumeris* AG-1 IC field isolates Rh28 and 1R4, their SBIs, and inter-F×B tuft isolates formed between the field isolates and their SBIs at 28°C. "F" indicates field isolate Rh28 or 1R4. Asterisk indicates SBI obtained from Rh28 or 1R4. "T" indicates inter-F×B tuft isolate formed between 1R4-SBIs and their non-parental field isolate Rh28-F, and between Rh28-SBIs and their non-parental field isolate 1R4-F. Values with the same letters are not significantly different according to Fisher's LSD test at *P*=0.05.

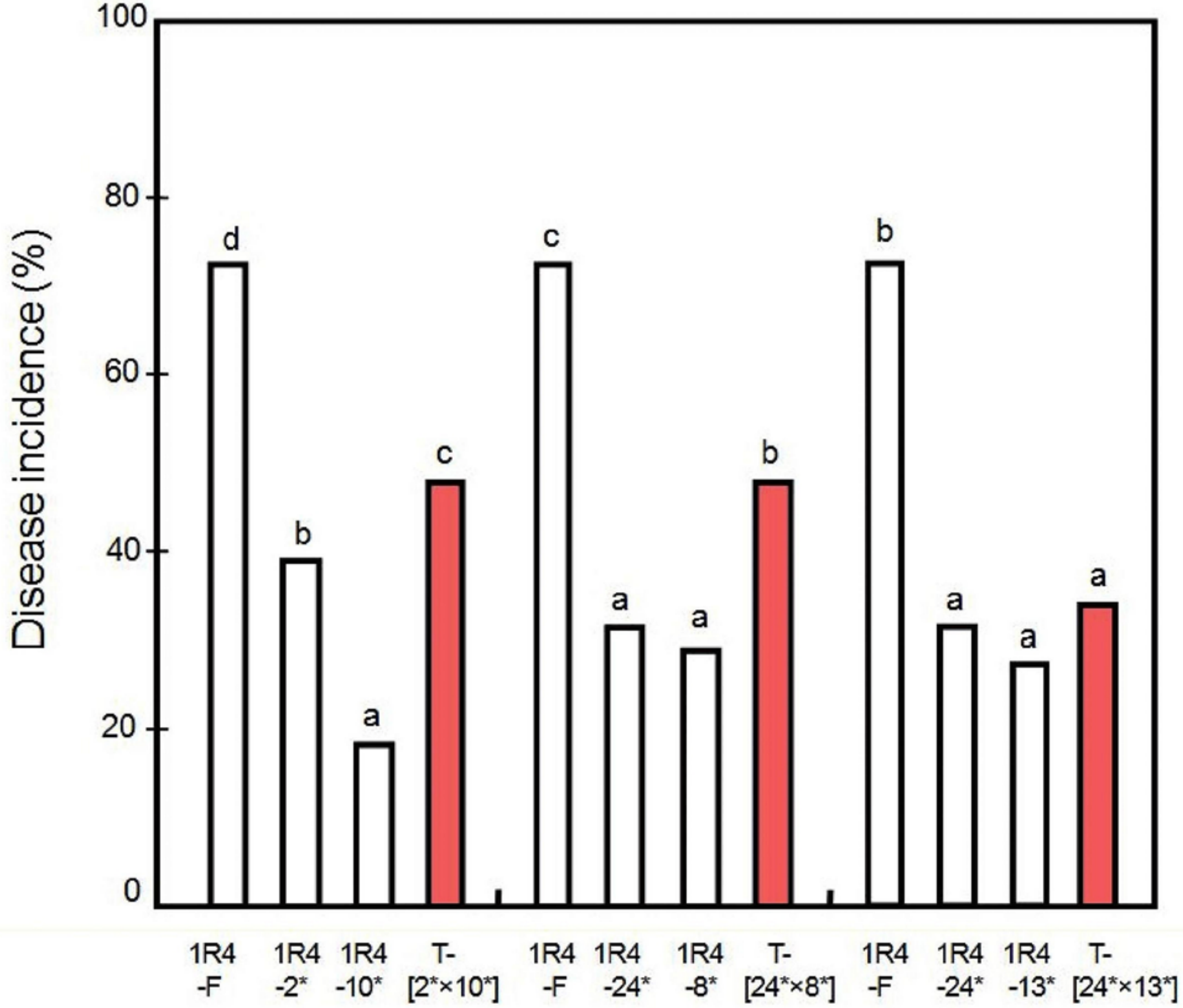

Figure S4. Disease incidence of *T. cucumeris* AG-1 IC field isolate 1R4, its SBIs and intra-B×B tuft isolates formed between SBIs-Mc1 and -Mc2. "F" indicates field isolate 1R4. Asterisk indicates SBI obtained from 1R4. "T" indicates intra-B×B tuft isolate formed between SBI-Mc1 and -Mc2 of 1R4. Values with the same letters are not significantly different according to Fisher's LSD test at P=0.05.

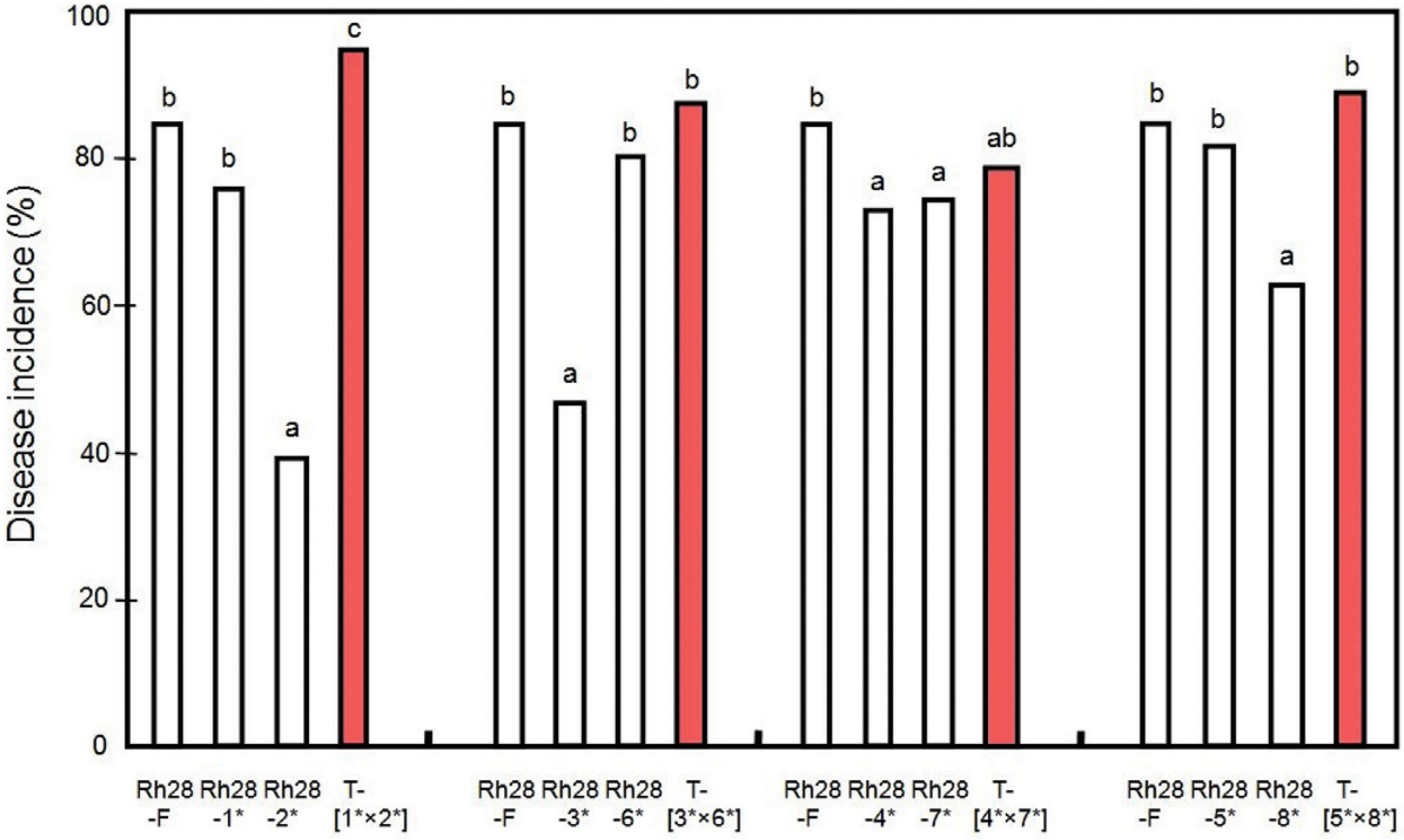

Figure S5. Disease incidence of *T. cucumeris* AG-1 IC field isolate Rh28, its SBIs and intra-B×B tuft isolates formed between SBIs-Mb1 and -Mb2. “F” indicates field isolate Rh28. Asterisk indicates SBI obtained from Rh28. “T” indicates intra-B×B tuft isolate formed between SBI-Mb1 and -Mb2 of Rh28. Values with the same letters are not significantly different according to Fisher’s LSD test at *P*=0.05.

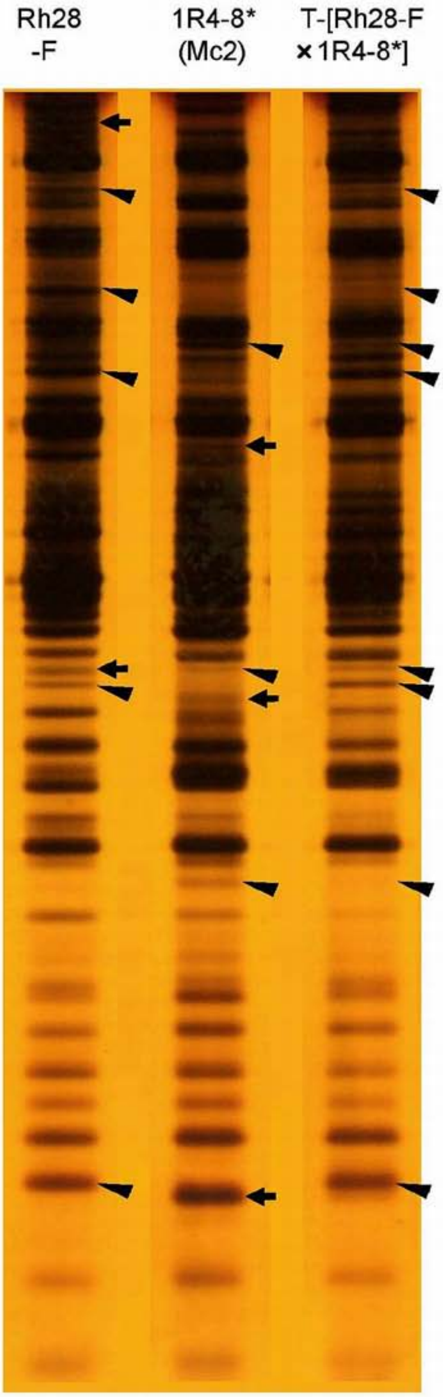

Figure S6. AFLP profile of the fingerprints of inter-F×B tuft isolate obtained from SBI 1R4-8* and field isolate Rh28 of *T. cucumeris* AG-1 IC. The primer pair used for selective amplifications was *Eco*RI-TG / *Mse*I-CA. Profile shows the difference among tuft isolate T-[Rh28-F×1R4-8*] and its contributing SBI 1R4-8* and field isolate Rh28-F. Triangles indicate the specific markers of each isolate. Arrows indicate specific markers present only in SBI 1R4-8* or field isolate Rh28-F.

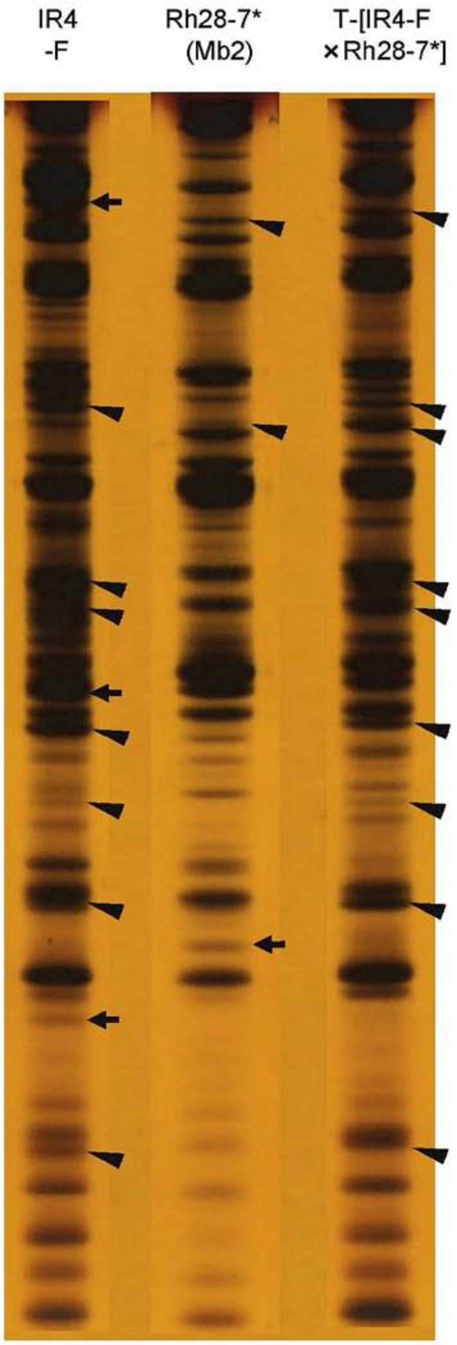

Figure S7. AFLP profile of the fingerprints of inter-F×B tuft isolate obtained from SBI Rh28-7* and field isolate 1R4 of *T. cucumeris* AG-1 IC. The primer pair used for selective amplifications was *Eco*RI-TG / *Mse*I-CA. Profile shows the difference among tuft isolate T-[1R4-F×Rh28-7*] and its contributing SBI Rh28-7* and non-parental field isolate 1R4. Triangles indicate the specific markers of each isolate. Arrows indicate specific markers present only in SBI Rh28-7* or field isolate 1R4.

Table S1. Types of tufts formed among single-basidiospore isolates and between SBIs and their parental/non-parental field isolates within *T. cucumeris* AG-1 IC and AG-2-2 IV

AG-1 IC										
		189[1)]			Rh28			1R4		
		F	Ma1[2)]	Ma2	F	Mb1	Mb2	F	Mc1	Mc2
189	F[3)]	-[4)]	NT[9)]	NT	-	NT	NT	-	inter-[6)] F×B	inter- F×B
	Ma1		-	intra-[5)] B×B[7)]	NT	inter- B×B	inter- B×B	inter- F×B	inter- B×B	inter- B×B
	Ma2			-	NT	inter- B×B	inter- B×B	inter- F×B	inter- B×B	inter- B×B
Rh28	F				-	intra- F×B	intra- F×B	-	NT	NT
	Mb1					-	NT	inter- F×B	inter- B×B	inter- B×B
	Mb2						-	inter- F×B	inter- B×B	inter- B×B
1R4	F							-	intra- F×B	intra- F×B
	Mc1								-	intra- B×B
	Mc2									-

AG-2-2 IV										
		SA-1			H10-28			H10-26		
		F	Mx1	Mx2	F	My1	My2	F	Mz1	Mz2
SA-1	F	-	intra- F×B	inter- F×B	-	NT	NT	-	inter- F×B	inter- F×B
	Mx1		-	intra- B×B	NT	inter- B×B	inter- B×B	NT	inter- B×B	inter- B×B
	Mx2			-	NT	inter- B×B	inter- B×B	NT	inter- B×B	inter- B×B
H10-28	F				-	intra- F×B	intra- F×B	-	inter- F×B	inter- F×B
	My1					-	intra- B×B	inter- F×B	inter- B×B	inter- B×B
	My2						-	inter- F×B	inter- B×B	inter- B×B
H10-268	F							-	intra- F×B	intra- F×B
	Mz1								-	intra- B×B
	Mz2									-

1): Single-basidiospore isolates obtained from each of the field isolates were grouped into mating types-1 and -2, respectively.

2): Mating type of SBIs obtained from each of the field isolates 189, Rh28 and 1R4 of AG-1 IC were assigned with the letters “a”, “b” and “c”; from each of the field isolates SA-1, H10-28 and H10-268 of AG-2-2 IV with the letters “x”, “y” and “z”, respectively.

3): Field isolate.

4): No tuft formation.

5): Tufts formed between isolates obtained from the same field isolates were assigned as “intra-”.

6): Tufts formed between isolates obtained from different field isolates were assigned as “inter-”.

7): Tufts formed between single-basidiospore isolates were assigned as “B×B”.

8): Tufts formed between parental/non-parental field isolates and single-basidiospore isolates were assigned as “F×B”.

9): Not tested.

Table S2. Tuft formation among single-basidiospore isolates and between SBIs and their parental/non-parental field isolates within *T. cucumeris* AG-1 IC and AG-2-2 IV, respectively.

		189[1]			Rh28			1R4		
		F	Ma1[2]	Ma2	F	Mb1	Mb2	F	Mc1	Mc2
189	F[3]	-[4]	+c[5]/+f*[6]	+c/+f*	-	+c/+f*	+c/+f*	-	+c/+f*	+c/+f*
	Ma1		-	+f[7]	+c/+f*	+f	+f	+c/+f*	+f	+f
	Ma2			-	+c/+f*	+f	+f	+c/+f*	+f	+f
Rh28	F				-	+c/+f*	+c/+f*	-	+c/+f*	+c/+f*
	Mb1					-	+f	+c/+f*	+f	+f
	Mb2						-	+c/+f*	+f	+f
1R4	F							-	+c/+f*	+c/+f*
	Mc1								-	+f
	Mc2									-

		SA-1			H10-28			H10-268		
		F	Mx1	Mx2	F	My1	My2	F	Mz1	Mz2
SA-1	F	-	+c	+c	-	+c	+c	-	+c	+c
	Mx1		-	+f	+c	+f	+f	+c	+f	+f
	Mx2			-	+c	+f	+f	+c	+f	+f
H10-28	F				-	+c	+c	-	+c	+c
	My1					-	+f	+c	+f	+f
	My2						-	+c	+f	+f
H10-268	F							-	+c	+c
	Mz1								-	+f
	Mz2									-

1): Single-basidiospore isolates obtained from each of the field isolates were grouped into mating types-1 and -2, respectively.

2): Mating type of SBIs obtained from each of the field isolates 189, Rh28 and 1R4 of AG-1 IC were assigned with the letters “a”, “b” and “c”; from each of the field isolates SA-1, H10-28 and H10-268 of AG-2-2 IV with the letters “x”, “y” and “z” respectively.

3): Field isolate.

4): No tuft (Sexual incompatibility).

5): Compact tuft (Sexual compatibility).

6): Sparse fibrous tuft (Sexual compatibility).

7): Fibrous tuft (Sexual compatibility).

Table S3. Tuft isolates produced by Hom-Hom and Het-Hom pairings among isolates of a common parent (intra-B×B and intra-F×B) of *T. cucumeris* AG-1 IC and AG-2-2 IV

	AG	Isolate	SCG group
Intra SBI □ SBI (Hom-Hom)	AG-1 1C	1R4-F, T1 [1*×6*], T11 [23*×25*]	SCG 1
		T8 [20*×12*]	SCG 2
		T9 [20*×22*], T10 [23*×22*]	SCG 3
		T2 [1*×7*], T3 [2*×10*], T4 [6*×12*], T5 [8*×24*],T6 [8*×25*], T7 [13*×24*]	SCG 4
	AG-2-2 IV	H10-28-F, T8-[13*×39*]	SCG 1
		T1-[7*×30*], T3-[7*×37*]	SCG 2
		T2-[7*×32*], T4-[7*×39*], T5-[13*×30*], T6-[13*×32*], T7-[13*×37*], T9-[25*×30*], T10-[25*×32*], T11-[25*×37*], T12-[25*×39*], T13-[27*×30*], T14-[27*×32*], T15-[27*×37*], T16-[27*×39*]	SCG 3
		H10-268-F, T9-[8*×2*], T11-[8*×12*]	SCG 1
		T8-[4*×15*]	SCG 2
		T2-[1*×7*], T10-[1*×2*]	SCG 3
		T1-[8*×7*], T3-[1*×12*], T4-[1*×15*], T5-[4*×2*], T6-[4*×7*], T7-[4*×12*], T12-[8*×15*], T13-[9*×2*], T14-[9*×7*]	SCG 4
Intra Parent isolate □ SBI (Het-Hom)	AG-1 1C	1R4-F, T2-[1R4-F×10*]	SCG 1
		T1-[1R4-F×2*], T3-[1R4-F×18*], T4-[1R4-F×23*]	SCG 2
	AG-2-2 IV	SA-1-F, T2-[SA-1-F×2*]	SCG 1
		T6-[SA-1-F×15*]	SCG 2
		T1-[SA-1-F×1*], T3-[SA-1-F×4*], T4-[SA-1-F×7*], T5-[SA-1-F×11*], T7-[SA-1-F×21*], T8-[SA-1-F×23*]	SCG 3
		H10-268-F, T6-[H10-268-F×9*]	SCG 1
		T2-[H10-268-F×2*], T5-[H10-268-F×8*]	SCG 2
		T1-[H10-268-F×1*], T3-[H10-268-F×4*], T4-[H10-268-F×7*], T7-[H10-268-F×10*], T8-[H10-268-F×11*], T9-[H10-268-F×12*], T10-[H10-268-F×15*]	SCG 3

Table S4. Tuft isolates produced by Hom-Hom and Het-Hom pairings among isolates of different parents (inter-B×B and inter-F×B) of *T. cucumeris* AG-1 IC and AG-2-2 IV

	AG	Isolate	SCG group
Inter SBI □ SBI (Hom-Hom)	AG-1 1C	189-F	SCG 1
		Rh28-F	SCG 2
		T1-[189-1*×Rh28-3*]	SCG 3
		T2-[189-1*×Rh28-7*]	SCG 4
		T3-[189-3*×Rh28-2*]	SCG 5
		T4-[189-3*×Rh28-7*], T6-[189-9*×Rh28-3*]	SCG6
		T5-[189-9*×Rh28-2*]	SCG 7
		T7-[189-10*×Rh28-3*]	SCG 8
		T8-[189-10*×Rh28-7*]	SCG 9
		189-F	SCG 1
		1R4-F	SCG 2
		T1-[189-1*×1R4-24*], T2-[189-1*×1R4-25*]	SCG 3
		T3-[189-3*×1R4-8*]	SCG 4
		T4-[189-3*×1R4-13*], T6-[189-3*×1R4-25*]	SCG 5
		T5-[189-3*×1R4-24*], T7-[189-9*×1R4-24*]	SCG 6
		T8-[189-10*×1R4-24*]	SCG 7
	AG-2-2 IV	SA-1-F	SCG 1
		H10-28-F	SCG 2
		T1-[SA-1-4*×H10-28-7*]	SCG 3
		T2-[SA-1-4*×H10-28-13*], T7-[SA-1-2*×H10-28-7*]	SCG 4
		T3-[SA-1-4*×H10-28-32*]	SCG 5
		T4-[SA-1-21*×H10-28-13*]	SCG 6
		T5-[SA-1-21*×H10-28-30*], T6-[SA-1-21*×H10-28-32*]	SCG 7
		T8-[SA-1-2*×H10-28-30*]	SCG 8
		T9-[SA-1-11*×H10-28-13*]	SCG 9
		T10-[SA-1-11*×H10-28-30*], T11-[SA-1-23*×H10-28-30*]	SCG 10
		SA-1-F	SCG 1
		H10-268-F	SCG 2
		T1-[SA-1-4*×H10-268-1*], T5-[SA-1-21*×H10-268-1*]	SCG 3
		T2-[SA-1-4*×H10-268-4*]	SCG 4
		T3-[SA-1-4*×H10-268-2*], T6-[SA-1-21*×H10-268-3*]	SCG 5
		T4-[SA-1-4*×H10-268-3*]	SCG 6
		T7-[SA-1-2*×H10-268-1*]	SCG 7
		T8-[SA-1-2*×H10-268-2*]	SCG 8
		T9-[SA-1-11*×H10-268-4*]	SCG 9
		T10-[SA-1-11*×H10-268-2*]	SCG 10
		T11-[SA-1-23*×H10-268-1*]	SCG 11

Table S4…Continuation…

	AG	Isolate	SCG group
Inter Parent isolate x SBI (Het-Hom)	AG-1 1C	1R4-F	SCG 1
		Rh28-F	SCG 2
		T1-[1R4-F×Rh28-1*]	SCG 3
		T2-[1R4-F×Rh28-2*]	SCG 4
		T3-[1R4-F×Rh28-3*]	SCG 5
		T4-[1R4-F×Rh28-7*]	SCG6
		1R4-F	SCG 1
		189-F	SCG 2
		T1-[1R4-F×189-1*]	SCG 3
		T2-[1R4-F×189-2*]	SCG 4
		T3-[1R4-F×189-3*]	SCG 5
		T4-[1R4-F×189-7*]	SCG 6
		T5-[1R4-F×189-9*]	SCG 7
		T6-[1R4-F×189-10*]	SCG 8
	AG-2-2 IV	H10-268-F	SCG 1
		H10-28-F	SCG 2
		T1-[H10-268-F×H10-28-7*]	SCG 3
		T2-[H10-268-F×H10-28-13*]	SCG 4
		T3-[H10-268-F×H10-28-25*]	SCG 5
		T4-[H10-268-F×H10-28-27*]	SCG 6
		T5-[H10-268-F×H10-28-30*]	SCG 7
		T6-[H10-268-F×H10-28-32*]	SCG 8
		T7-[H10-268-F×H10-28-37*], T8-[H10-268-F×H10-28-39*]	SCG 9
		SA-1-F	SCG 1
		H10-268-F	SCG 2
		T1-[SA-1-F×H10-268-1*]	SCG 3
		T2-[SA-1-F×H10-268-2*]	SCG 4
		T3-[SA-1-F×H10-268-3*]	SCG 5
		T4-[SA-1-F×H10-268-4*]	SCG 6
		T5-[SA-1-F×H10-268-7*]	SCG 7
